# Assessment of the role of gut health in childhood stunting in a multisite, longitudinal study in India, Indonesia and Senegal: a UKRI GCRF Action Against Stunting Hub protocol

**DOI:** 10.1136/bmjpo-2022-001637

**Published:** 2024-02-27

**Authors:** Benjamin Momo Kadia, Anouschka S Ramsteijn, Teena Dasi, Umi Fahmida, Bharati Kulkarni, Babacar Faye, Min Kyaw Htet, Doudou Sow, Rajender Rao Kalashikam, Ritu Sharma, Arienta R P Sudibya, Sari Kusuma, Tiffany C Angelin, Mifa Nurfadilah, Modou Lamin Jobarteh, Ndeye Sokhna Diop, Isobel Gabain, Beatriz Calvo-Urbano, Elaine Ferguson, Paul Haggarty, Claire Heffernan, Joanne P Webster, Alan W Walker, Stephen Allen

**Affiliations:** 1 Department of Clinical Sciences, Liverpool School of Tropical Medicine, Liverpool, UK; 2 Rowett Institute, University of Aberdeen, Aberdeen, UK; 3 ICMR-National Institute of Nutrition, Hyderabad, India; 4 Southeast Asian Ministry of Education Organisation Regional Centre for Food and Nutrition (SEAMEO RECFON), East Jakarta, Indonesia; 5 Service de Parasitologie-Mycologie, Faculté de Médecine, Université Cheikh Anta Diop (UCAD), Dakar, Sénégal; 6 Service de Parasitologie-Mycologie, UFR Sciences de la Santé, Université Gaston Berger, Saint Louis, Sénégal; 7 Department of Population Health, London School of Hygiene & Tropical Medicine, London, UK; 8 Department of Pathobiology and Population Sciences, Royal Veterinary College, University of London, London, UK; 9 London International Development Centre, London, UK

**Keywords:** gastroenterology, growth, microbiology, molecular biology, parasitology

## Abstract

**Introduction:**

Childhood stunting has a complex aetiology, with poor gut health being an important contributor. This study will assess inter-relationships between maternal and infant gut health indices and infant linear growth. Inter-relationships between gut health indices, systemic inflammation and growth hormones in early childhood will also be assessed.

**Methods and analysis:**

A longitudinal observational study of cohorts of 600 newborns and their mothers in India, Indonesia and Senegal will be conducted. Women will be recruited during pregnancy and their children followed up to age 24 months. Stool, urine and blood samples will be collected from the women and children for assessments of helminthic and protozoal parasites, bacterial pathogens, faecal microbiota taxa, biomarkers of environmental enteric dysfunction, systemic inflammation and growth hormones. Child anthropometric measurements will be collected at birth and at ages 3, 6, 9, 12, 18 and 24 months. The gut health indices will be integrated with cohort data from other Action Against Stunting Hub (AASH) workstreams for interdisciplinary analyses of childhood stunting and the development of a new typology of stunting.

**Discussion:**

This study will advance scientific understanding of the role of gut health in childhood stunting and will contribute to a broader knowledge of the complex aetiology of this condition as part of the interdisciplinary AASH research to reduce the global burden of childhood stunting.

**Ethics and dissemination:**

This study has been approved by the relevant Ethics Committees in Senegal, India, and Indonesia and LSHTM. The results will be submitted for publication in peer-reviewed journals.

**WHAT IS ALREADY KNOWN ON THIS TOPIC**

- In areas with poor hygiene and sanitation, recurrent enteric infections and alterations in the gut microbiota are associated with environmental enteric dysfunction (EED).
- EED is an important precursor of childhood stunting.

**WHAT THIS STUDY ADDS**

- Compare characteristics of gut health in mothers and their infants and elucidate their association with childhood stunting across three distinct geographical settings, with the inclusion of mothers being a novel approach providing more robust evidence on early life determinants of childhood stunting.
- Contribute to the development of a new typology of childhood stunting based on knowledge of the relationship between the gut and other host and environmental factors, and to support development of interdisciplinary approaches to prevent and reduce childhood stunting.

**HOW THIS STUDY MIGHT AFFECT RESEARCH, PRACTICE OR POLICY**

- Identify biomarkers of gut health that may serve as outcome measures in future interventional studies and inform the development of diagnostic and management options for EED in resource-constrained settings.

## Introduction

Worldwide, more than one in five children under 5 years of age are currently stunted, with more than half residing in Asia and two out of five in Africa.^1^ Childhood stunting is defined as having a height-for-age z-score more than 2 standard deviations below the median of the WHO child growth standards.^2^ While the aetiology of stunting is complex, several studies suggest that it is associated with environmental enteric dysfunction (EED) resulting from asymptomatic gut infection with pathogenic microbes.^3 4^

EED refers to the abnormal gut structure and function commonly found in people living in environments with poor sanitation and hygiene, with onset detected as early as 12 weeks after birth despite exclusive breast feeding.^5 6^ EED is characterised by villous atrophy, crypt hyperplasia, chronic enteric inflammation and increased gut mucosal permeability. It leads to reduced nutrient absorption, increased risk of systemic infection due to reduced integrity of the gut barrier, particularly due to gut-derived Gram-negative bacteria, and impaired immune responses to oral vaccines.^5 6^ EED contributes to stunting through nutrient malabsorption and systemic inflammation, with reduced production of growth hormones.^5–7^ Furthermore, EED has been associated with disruption in the ecological niches that support the gut microbiota.^5^ It is known that malnutrition and growth faltering in early life are associated with microbiota immaturity,^8^ and experimental evidence suggests that the gut microbiota may be causally involved in the manifestation of stunting.^9^ The gut microbiota also plays an important role in protecting against colonisation and invasion of the gut by pathogens.^6^

Previous studies of stunting have explored various biomarkers of EED, including plasma α_1_-acid glycoprotein (AGP, a longer-term marker of systemic inflammation),^7 10–15^ faecal myeloperoxidase (MPO, a marker of intestinal inflammation),^11–13 16^ faecal α_1_-antitrypsin (AAT, a marker of protein loss and intestinal permeability)^11 13 16^ and plasma intestinal fatty acid binding protein (IFABP, a marker of mucosal permeability and gut integrity),^7 10 16 17^ and also measured plasma growth hormones (insulin-like growth factor (IGF)-1 and its binding protein IGFBP3).^7 10 12^ However, a consensus has not been reached on the biomarkers to use as standard for diagnosing and monitoring EED. Thus, in low-income settings where EED and stunting are most prevalent, the association of biomarkers of EED with linear growth requires further investigation.

In this study, we will assess aspects of gut health, including gut microbiota profiles, colonisation of the gut by pathogenic microbes (bacterial and parasitic pathogens), and biomarkers of EED and associated pathology (systemic inflammation and reduced growth hormones) during pregnancy and early childhood, and their role in childhood stunting.

## Methods and analysis

### Study design and population

This is a longitudinal, observational study which is part of the UKRI GCRF Action Against Stunting Hub (AASH). Full details of the overall AASH design are available in another section of this UKRI GCRF Action Against Stunting Hub supplement (Jobarteh ML *et al.* Developing a ‘Whole Child Approach’ to understanding and preventing child stunting: the UKRI GCRF Action Against Stunting Hub*. BMJ Paed. Open* 2023), but details relating to the Gut Health component workstream are summarised below.

Women in the second and third trimesters of pregnancy will be recruited in Hyderabad in India (urban), East Lombok in Indonesia (rural) and Kaffrine District in Senegal (rural), and their newborns will be followed up to age 24 months. These three areas were chosen because they have high proportions of stunted growth. In India, 30.9% of children under 5 are stunted^1^ and Hyderabad is among the highest burden districts, bearing up to 11.6% of all stunted children under five in the country.^18^ In Indonesia, 31.8% of children under 5 are stunted,^1^ with East Lombok having one of the highest subnational rates of about 44%.^19^ Despite having a lower prevalence (17.2%), Senegal is still off-track to achieve a 50% reduced prevalence of stunted growth by 2030.^1^ In Kaffrine district, up to 26% of children under 5 are stunted, which exceeds the national prevalence.^20^

For recruitment purposes, the sampling frame (lists of pregnant women) will be obtained from health services in each country. From the lists, 50 women will be randomly selected each month and those who meet the inclusion criteria will be recruited after they provide written informed consent (supplemental file 1). If women from this random selection do not fit the inclusion criteria, women will be selected from the rest of the initial lists for that month.

### Sample size determination

Sample size calculations were performed in R with the package ‘pwr’ and were based on a multiple linear regression analysis with 10 predictors, an effect size of 0.035, power of 80%, significance level of 0.05 and drop-out rate of 20%. These calculations resulted in a total of 592 participants per site.

### Study procedures

#### Sample collection

Stool samples will be collected from pregnant women in the third trimester. In infants/children, stool samples will be collected either at a health facility or in the participants’ homes at ages 1 month, 6 months and 24 months. For stool samples collected at home, study staff will visit either on the day before or on the morning of sample collection to provide the mother/carer with a disposable nappy or plastic sheet, sample collection containers with spatula, plastic specimen bag, small cold box with ice pack and a bar of soap. The staff will explain the sample collection procedure to the mother/carer through verbal and pictorial information, and the staff will check the procedure is understood. Mothers/carers will be asked to snugly fit a nappy or plastic sheet to the infant last thing at night and collect the next stool produced by the infant, either by pouring it from the nappy/sheet if the sample is loose, or using the spatula if it is formed, and fill the sample containers. They will place the filled container in the specimen bag and then in the cold box and wash their hands with soap. Field staff will return later the same day or the following day to label the sample and transfer it to the laboratory. Subject to agreement with the mother/carer, this process will be repeated over the following 14 days until an adequate stool sample is obtained (at least 5 mL of liquid stool or 5 g of formed stool). To avoid dilution of biomarkers in children suffering from acute diarrhoea, stool collection will be delayed until the stools have returned to their usual consistency. Based on the WHO definition,^21^ acute diarrhoea will be considered as significantly more frequent or loose/watery stools in the mother’s/carer’s opinion in exclusively breastfed babies and three or more liquid stools in the last 24 hours in children receiving other feeds. Mothers/carers will be fully briefed on recognising acute diarrhoea as distinct from loose stools within exclusively breastfed babies. During the visits to collect samples at age 6 and 24 months, trained research staff will collect 2.5 mL venous blood. Subject to the agreement of the mother/carer, efforts will be made to limit attempts to collect the required blood volume to three. Finally, early morning urine samples will be collected from pregnant mothers and infants at 6 and 24 months in Senegal to identify the presence and intensity of *Schistosoma haematobium* and hybrids therein. All samples will be transported to laboratories in a cold box. The time between sample collection and receipt in the laboratory will be recorded. Pilot testing of sample collection processes was conducted in each country.

Anthropometric measurements (including length and weight) will be taken in duplicate from infants at birth, 3, 6, 9, 12 18 and 24 months of age as described in detail in another protocol in this UKRI GCRF Action Against Stunting Hub supplement (Davies-Kershaw H *et al.* Anthropometric, biochemical, dietary, morbidity and well-being assessment in women and children in Indonesia, India and Senegal: a UKRI GCRF Action Against Stunting Hub protocol. *BMJ Paed. Open* 2023). Z-scores for length-for-age (LAZ) will be calculated using the 2006 WHO Growth Standards and stunted growth will be defined as an LAZ <-2 SD.^2^

#### Laboratory analysis

Approximately 1 g of faecal material will be aliquoted into a DNA/RNA Shield Faecal Collection Tube containing a preservative buffer solution (Zymo Research, California, USA) for DNA extraction using the ZymoBIOMICS DNA Miniprep kit (Zymo Research, California, USA) for subsequent microbiome and qPCR-based analyses. Stool samples and aliquots in DNA/RNA Shield will be stored at −20°C until further processing.

Kato-Katz thick smear microscopy (duplicate slides per single stool) will be performed on fresh (untreated) stool to obtain individual egg counts of key helminth parasites reported and/or predicted to be associated with physical and/or cognitive childhood stunting (eg, *Ascaris* spp., hookworm, *Trichuris trichiura, Hymenolepis nana*).^22^ Slides will be read within 60 min post Kato-Katz preparation to obtain hookworm data. The slides will then be maintained at room temperature prior to microscopic examination of the remaining parasites present. The amount of stool placed on the Kato Katz slide is 41.7 mg, so the numerical values obtained will be multiplied by 24 to give the number of eggs per gram (epg)/Faecal Egg Count—the standard measurement to assess the intensity of infection.

Multiparallel qPCR on DNA extracted from faeces will be performed for high sensitivity and specificity identification of key gastrointestinal parasites,^23^ based on a modified protocol with updated primers and quality control.^24 25^ This is a high throughput, species-specific molecular diagnostic tool for routine surveillance, monitoring and research, which allows more accurate identification and quantification of key pathogens associated with physical and cognitive stunting in children relative to slide-based microscopy alone.^24 25^ Infection levels can be more precisely measured in low prevalence settings where intensities are low (as predicted in infants). This will target *Ascaris lumbricoides*, *Ancylostoma duodenale*, *Necator americanus*, *Trichuris trichiura*, *Strongyloides stercoralis* and protozoans such as *Giardia lamblia*, *Cryptosporidium parvum/hominis* and *Entamoeba histolytica*/*dispar*.

Multiplex PCR will also be used to identify pathogenic strains of *Escherichia coli*: enteropathogenic *E. coli*, enteroaggregative *E. coli*, enterotoxigenic *E. coli* and enteroinvasive *E. coli* using methods and primers developed for the Malnutrition and Enteric Disease study.^26^ In addition, analysis of the faecal microbiota (see below) may also putatively identify potential enteropathogens at the genus level, such as *Helicobacter, Campylobacter*, *Plesiomonas*, *Yersinia*, *Aeromonas, Shigella/Escherichia* and *Salmonella* spp.

For microbial culture to detect *Salmonella* spp. and *Shigella* spp., swabbing onto agar plates or inoculating approximately 1 g of fresh stool in Selenite broth for enrichment followed by culture in selective media MacConkey Agar and Xylose Lysine Deoxycholate Agar or by automated culture in Senegal will be done immediately after samples are brought to the laboratory. Gram staining and biochemical tests such as Kligler Iron Agar, Motility Indole Ornithine, Lysine decarboxylase, Urea and Oxidase will be used for identifying the cultured isolates at the genus level, complemented by serological tests to identify the bacterial species using BD Difco Salmonella O Antiserum Poly A-I and BD Difco Shigella Antiserum Poly Group A, A1, B, C, C1, C2 and D.

MPO and alpha 1-antitrypsin will be measured in stool samples using the Immunodiagnostik AG (Bensheim, Germany) ELISA Kit. Sample preparation and analysis will be as per manufacturer’s instructions.

Microbiota profiling will be done by 16S rRNA gene amplicon sequencing. We will use barcoded primers from the Earth Microbiome Project protocol,^27^ with slight modifications to increase detected diversity,^28^ to amplify the V4 region of the 16S rRNA gene using PCR on DNA extracted from stool samples. 16S rRNA gene amplicon sequencing will be carried out using the Illumina MiSeq platform. For amplicons from Indonesia and Senegal this will be carried out by sequencing providers in the UK, while sequencing of Indian sample amplicons will take place at a sequencing provider in India. Negative (no DNA template ‘blanks’) and positive (extracted DNA and 16S rRNA gene amplicons from one benchmark stool sample, and from a microbial community standard) control samples will be included in each sequencing run for quality control purposes, and to ensure consistency between runs, both longitudinally and between participant countries.

For the urine samples in Senegal, assessment for blood in urine will be performed immediately on receipt in the laboratory (Hemastix Reagent Strip; Bayer, Diagnostics Div., New York, USA). Standard urine filtration and microscopy (duplicate 10 mL per urine sample) will be performed to identify and quantify *S. haematobium* (and hybrids therein) infection status and recorded as eggs per 10 mL/urine. Positive filters will be placed in fresh water and exposed to light to facilitate egg-hatching into miracidia. Free-swimming miracidia will be individually pipetted onto Whatman Indicating FTA Classic cards (GE Healthcare Life Sciences, UK) for DNA storage and subsequent molecular analysis. Individual *Schistosoma* DNA extracts will be characterised by amplification of a partial fragment of the mitochondrial cytochrome c oxidase subunit 1 (*cox1*) and the complete nuclear ribosomal DNA internal transcribed spacer to identify species/species-combination, following protocols described elsewhere.^29^

Blood samples will be centrifuged and separated on receipt at the laboratory and plasma stored at −80°C until analyses. Samples in Senegal will be stored at −20°C and transported to Dakar within days of collection to store at −80°C. Blood samples will be analysed for biomarkers of inflammation (AGP; Human AGP Quantikine ELISA Kit) and C reactive protein (CRP; Human CRP/CRP Quantikine ELISA kit in India and Senegal; Quansys multiplex in Indonesia); gut mucosal integrity (IFABP; Human FABP2/I-FABP Quantikine ELISA Kit in India and Senegal; Quansys multiplex in Indonesia) and plasma growth hormones (IGF-1 and IGF BP3; Human IGF-1 and IGFBP-3 Quantikine ELISA Kits respectively, R&D Systems, Minneapolis, USA in India and Senegal and Quansys multiplex in Indonesia). Assays will be carried out according to the manufacturers’ instructions at the National Institute of Nutrition, Hyderabad in India and the Université Cheikh Anta DIOP, Dakar in Senegal. In Indonesia, sample preparation will be done in Lombok and analyses in SEAMEO RECFON’s laboratory in Jakarta.

All participants with clinically relevant bacterial or parasitic infections will be offered treatment. After these analyses, sample aliquots will be stored at −80°C.

### Data management and analysis

Data management will follow procedures established by the AASH (see Jobarteh ML *et al.* Developing a ‘Whole Child Approach’ to understanding and preventing child stunting: the UKRI GCRF Action Against Stunting Hub. *BMJ Paed. Open* 2023). This includes a data sharing strategy that will build on the guiding principles for scientific management and stewardship and Concordat on Open Research Data to ensure equitable access to data.

For microbiota analysis, FASTQ files containing 16S rRNA gene amplicon sequences will be processed using well-validated software pipelines such as mothur^30^ or DADA2.^31^ These pipelines include various quality control steps and will produce final output tables containing the proportional abundances of various microbiota taxa in each sample. They can also generate various metrics of bacterial diversity within and between samples. These data will be visualised in the form of dendrograms, ordination plots, heatmaps and box plots. Microbiota 16S rRNA gene sequence data will be released publicly via the European Nucleotide Archive at the end of the study.

Multilevel models taking into consideration aggregate units such as geographical setting will be used to investigate associations between enteropathogens/gut parasites, biomarker concentrations and LAZ, to assess longitudinal associations between biomarker concentrations and growth rates, and to determine which biomarkers, enteropathogens and/or characteristics of the gut microbiota are associated with stunted growth at specific time points. In addition, Bayesian methods, using directed acyclic graphs, will be used to investigate a variety of possible interactions between gut health indices and linear growth (stunting) and to assess combinatorial effects of the diverse elements of gut health (expressed as categorical or continuous variables, as appropriate) on stunting. When the data being analysed include repeated measurements from the same individual, random intercept models will be employed. The number of missing data points for each variable of interest will be reported and the distributions of key variables will be tabulated to compare individuals with and without complete data. Analyses will be carried out both on datasets where multiple imputation will be implemented, and on datasets that will be restricted to complete cases.

To allow for interdisciplinary analyses and the development of the typology of stunting, multilevel modelling and Bayesian methods will be extended to integrate cohort data collected in other workstreams. Stata V.17 and other appropriate software will be used for the analysis.

### Patient and public involvement

There was no involvement of patients or the public in the design of this study.

## Discussion

The limited impact of interventions to prevent or ameliorate stunting in young children in low-resource settings has resulted in a renewed interest in the role of gut health. The AASH cohorts will be among the largest investigating the inter-relationships between gut colonisation with bacterial, protozoal and helminth pathogens, the gut microbiota and biomarkers of EED, and their impact on linear growth in populations with different prevalence rates of stunting (moderate to high) and environmental conditions.

The AASH offers the opportunity to compare gut health characteristics and their association with stunting across three distinct geographical settings. This is particularly important as there is limited evidence to date regarding the role of gut health in children in West Africa and Southeast Asia. In addition, our findings will be integrated with data collected in other AASH workstreams to advance scientific understanding of how gut health interacts with other host and environmental factors that underlie common typologies of stunting and increase the evidence base for future intervention studies. This evidence base will be further strengthened by the investigation of elements of maternal gut health as novel drivers of poor gut health and stunted growth in infants. Harmonised procedures to collect, manage and analyse data and samples across the countries will greatly enhance the validity of our findings.

In low-income settings, there is a need to further investigate biomarkers of intestinal structure and function to identify acceptable and affordable tools that can be scaled up at the population level to diagnose and monitor EED. Further, it is of interest to identify possible geographic-specific and seasonal variations in the association between these biomarkers and stunting. The selection of biomarkers for this study was informed by previous reports of their utility in reflecting the aetiopathogenesis of EED and association with linear growth faltering.^10 12 13 15 16^

This study will have some limitations. Although our study design allows us to explore similarities and differences across cohorts from three countries, our findings may not be generalisable to gut health and growth in other low-resource settings where the intensity of infection, characteristics of the gut microbiota and pathology of EED may differ. In general, it is a challenge to move from associations to an understanding of causality in an observational study. However, the SENeGal SYNbiotic (SENGSYN) study, a clinical trial nested within the AASH investigating the effect of supplementation with synbiotics on growth and biomarkers of gut health (seeMomo Kadia B *et al*. Improving gut health and growth in early life: a protocol for an individually randomised, two-arm open-label, controlled trial of a synbiotic in infants in Kaffrine District, Senegal. *BMJ Paed Open* 2023), may build on associations between gut health and growth determined in the observational cohorts to provide evidence of causality. From a technical perspective, our method of stool collection using cold boxes and minimising time between sample provision and processing in the laboratories is pragmatic. However, we cannot exclude the possibility that changes in stool biomarkers, pathogens and the microbiota may occur during transfer. We will, however, assess whether time between collection and receipt in the laboratory is associated with the results of the various analyses. We also acknowledge that providing a picture-based reference chart for mothers/carers to assess stool consistency would be an even more robust approach to assess stool consistency for the exclusion of diarrhoeal samples. Nonetheless, as part of quality control, trained laboratory technicians will cross-reference all collected samples to ensure they are of appropriate consistency before being further processed. Furthermore, we acknowledge that the WHO Child Growth Standards do not necessarily provide a good statistical fit to each country, but these internationally approved standards are widely accepted and facilitate cross-country comparisons, which is considered particularly important when including the three countries in this study. Previous multicountry studies, notably the Malnutrition and Enteric Disease study study,^2^ have used the same growth standards.

## Conclusions

The findings of this study will improve scientific understanding of how gut health contributes to stunting, either directly, or through interlinks with the wide range of host- and environmental factors (including maternal gut health characteristics) monitored in the AASH project, and how these may vary between different geographical locations. Our findings will inform the development of diagnostic- and management options for EED and identify biomarkers of gut health that may serve as outcome measures in future intervention trials in resource-constrained settings.

10.1136/bmjpo-2022-001637.supp1 Supplementary data

## Supplementary Material

Author's manuscript

## Data Availability

No data are available. Data sharing is not applicable to this manuscript as it describes a protocol.
