# Supplementary material for: Assessment of the role of gut health in childhood stunting in a multisite, longitudinal study in India, Indonesia and Senegal: a UKRI GCRF Action Against Stunting Hub protocol
Source: BMJ Paediatr Open. 2024 Feb 27;8(Suppl 1):e001637. doi: 10.1136/bmjpo-2022-001637 (PMC10900321; doi:10.1136/bmjpo-2022-001637)
Supplement: Supplementary data [file bmjpo-2022-001637supp001.pdf]

PARTICIPANT CONSENT FORM

Title of Project: UKRI GCRF Action against Stunting Hub – Observational Cohort Study - Mothers
Name of PI/Researcher responsible for project: Professor Claire Heffernan

**Statement**	**Please initial or thumbprint* each box**
I confirm that I have read the information sheet dated 26/11/2019 (version 2) for the above named study. I have had the opportunity to consider the information, ask questions and have these answered satisfactorily.<br>**OR**<br>I have had the information explained to me by study personnel in a language that I understand. I have had the opportunity to consider the information, ask questions and have these answered satisfactorily.	
I understand that my participation is voluntary and that I am free to withdraw at any time without giving any reason, without my/my baby's medical care or legal rights being affected.	
I understand that relevant sections of my/my baby's medical notes and data collected during the study may be looked at by authorised individuals from the study team, where it is relevant to my/my baby taking part in this research. I give permission for these individuals to have access to my records. I understand that they will only access information relevant to the birth of my baby.	
I understand that data about me/my baby may be shared via a public data store or by sharing directly with other researchers, and that I/my baby will not be identifiable from this information.	
I understand that the samples of blood, saliva, urine, hair, poo and breastmilk collected from me/my baby may be used to support other research in the future, and may be shared anonymously with other researchers, for their ethically-approved projects.	
I understand and agree that my words may be quoted in publications, reports, web pages, and other research outputs. I understand that I will not be named or in any way personally identifiable in these outputs.	
I understand that I may be contacted at a later date to consider participating in a separate study about my baby's health.	
I agree to take part in the above named study.	

Printed name of participant	Signature of participant	Date

Printed name of impartial witness*	Signature of impartial witness*	Date

I attest that I have explained the study information accurately in ________________ to, and was understood to the best of my knowledge by, the participant and that he/she has freely given their consent to participate* in the presence of the above named impartial witness (where applicable).

Printed name of person obtaining consent	Signature of person obtaining consent	Date

[*Only required if the participant is unable to read or write.]

A copy of this informed consent document has been provided to the participant.

Centre Number:
Study Number:
Participant Identification Number:

[Informed Consent for Participant with Impartial witness_26.11.19_v2]
