# Supplementary material for: Assessment of the role of gut health in childhood stunting in a multisite, longitudinal study in India, Indonesia and Senegal: a UKRI GCRF Action Against Stunting Hub protocol
Source: BMJ Paediatr Open. 2024 Feb 27;8(Suppl 1):e001637. doi: 10.1136/bmjpo-2022-001637 (PMC10900321; doi:10.1136/bmjpo-2022-001637)
Supplement: Author's manuscript [file bmjpo-2022-001637.draft_revisions.pdf]

BMJ Paediatrics Open

BMJ Paediatrics Open is committed to open peer review. As part of this commitment we make the peer review history of every article we publish publicly available.

When an article is published we post the peer reviewers' comments and the authors' responses online. We also post the versions of the paper that were used during peer review. These are the versions that the peer review comments apply to.

The versions of the paper that follow are the versions that were submitted during the peer review process. They are not the versions of record or the final published versions. They should not be cited or distributed as the published version of this manuscript.

BMJ Paediatrics Open is an open access journal and the full, final, typeset and author-corrected version of record of the manuscript is available on our site with no access controls, subscription charges or pay-per-view fees (http://bmjpaedsopen.bmj.com).

If you have any questions on BMJ Paediatrics Open's open peer review process please email info.bmjpo@bmj.com

BMJ Paediatrics Open

Assessment of the role of gut health in childhood stunting in a multi-site, longitudinal study in India, Indonesia, and Senegal: a UKRI GCRF Action Against Stunting Hub protocol paper

Journal:	*BMJ Paediatrics Open*
Manuscript ID	bmjpo-2022-001637
Article Type:	Protocol
Date Submitted by the Author:	08-Aug-2022
Complete List of Authors:	Momo Kadia, Benjamin; Liverpool School of Tropical Medicine Department of Clinical Sciences<br>Ramsteijn, Anouschka; University of Aberdeen<br>Dasi, Teena; National Institute of Nutrition<br>Fahmida, Umi; SEAMEO Regional Center for Food and Nutrition<br>Kulkarni, Bharati; National Institute of Nutrition<br>Faye, Babacar; Cheikh Anta Diop University of Dakar<br>Htet, Min Kyaw; SEAMEO Regional Center for Food and Nutrition<br>Sow, Doudou; Université Gaston Berger<br>Kalashikam, Rajender Rao; National Institute of Nutrition<br>Sharma, Ritu; National Institute of Nutrition<br>Sudibya, Arienta R.P.; SEAMEO Regional Center for Food and Nutrition<br>Kusuma, Sari; SEAMEO Regional Center for Food and Nutrition<br>Angelin, Tiffany C.; SEAMEO Regional Center for Food and Nutrition<br>Nurfadilah, Mifa; SEAMEO Regional Center for Food and Nutrition<br>Jobarteh, Modou; London School of Hygiene & Tropical Medicine<br>Diop, Ndeye Sokhna; Cheikh Anta Diop University of Dakar<br>Gabain, Isobel; University of London, Department of Pathobiology and Population Sciences<br>Calvo-Urbano, Beatriz; University of London, Department of Pathobiology and Population Sciences<br>Ferguson, Elaine; London School of Hygiene & Tropical Medicine<br>Haggarty, Paul; University of Aberdeen<br>Heffernan, Claire; London School of Hygiene & Tropical Medicine; London International Development Centre<br>Webster, Joanne P.; University of London, Department of Pathobiology and Population Sciences<br>Walker, Alan; University of Aberdeen<br>Allen, Stephen; Liverpool School of Tropical Medicine Department of Clinical Sciences
Keywords:	Gastroenterology, Growth, Microbiology, Molecular Biology, Parasitology

SCHOLARONE™
Manuscripts

I, the Submitting Author has the right to grant and does grant on behalf of all authors of the Work (as defined in the below author licence), an exclusive licence and/or a non-exclusive licence for contributions from authors who are: i) UK Crown employees; ii) where BMJ has agreed a CC-BY licence shall apply, and/or iii) in accordance with the terms applicable for US Federal Government officers or employees acting as part of their official duties; on a worldwide, perpetual, irrevocable, royalty-free basis to BMJ Publishing Group Ltd ("BMJ") its licensees and where the relevant Journal is co-owned by BMJ to the co-owners of the Journal, to publish the Work in this journal and any other BMJ products and to exploit all rights, as set out in our licence.

The Submitting Author accepts and understands that any supply made under these terms is made by BMJ to the Submitting Author unless you are acting as an employee on behalf of your employer or a postgraduate student of an affiliated institution which is paying any applicable article publishing charge ("APC") for Open Access articles. Where the Submitting Author wishes to make the Work available on an Open Access basis (and intends to pay the relevant APC), the terms of reuse of such Open Access shall be governed by a Creative Commons licence – details of these licences and which Creative Commons licence will apply to this Work are set out in our licence referred to above.

Other than as permitted in any relevant BMJ Author's Self Archiving Policies, I confirm this Work has not been accepted for publication elsewhere, is not being considered for publication elsewhere and does not duplicate material already published. I confirm all authors consent to publication of this Work and authorise the granting of this licence.

Confidential: For Review Only

Assessment of the role of gut health in childhood stunting in a multi-site, longitudinal study in India, Indonesia, and Senegal: a UKRI GCRF Action Against Stunting Hub protocol paper

Benjamin Momo Kadia[1], Anouschka S. Ramsteijn[2], Teena Dasi[3], Umi Fahmida[4], Bharati Kulkarni[3], Babacar Faye[5], Min Kyaw Htet[4], Doudou Sow[6], Rajender Rao Kalashikam[3], Ritu Sharma[3], Arienta R.P. Sudibya[4], Sari Kusuma[4], Tiffany C. Angelin[4], Mifa Nurfadilah[4], Modou L. Jobarteh[7], Ndeye Sokhna Diop[5], Isobel Gabain[8], Beatriz Calvo-Urbano[8], Elaine Ferguson[7], Paul Haggarty[2], Claire Heffernan[7,8,9], Joanne P. Webster[8], Alan W. Walker[2α], Stephen J Allen[1α]

[α]Contributed equally

Authors' affiliations

1. Department of Clinical Sciences, Liverpool School of Tropical Medicine (LSTM), Liverpool, United Kingdom (UK)
2. Rowett Institute, University of Aberdeen, Aberdeen, UK
3. National Institute of Nutrition, Hyderabad, India
4. Southeast Asian Ministry of Education Organisation Regional Centre for Food and Nutrition (SEAMEO RECFON), East Jakarta, Indonesia
5. Service de Parasitologie-Mycologie, Faculté de Médecine, Université Cheikh Anta Diop (UCAD), Dakar, Sénégal
6. Service de Parasitologie-Mycologie, UFR Sciences de la Santé, Université Gaston Berger, Saint Louis, Sénégal
7. London School of Hygiene and Tropical Medicine (LSHTM), London, UK
8. Department of Pathobiology and Population Sciences, Royal Veterinary College, University of London, London, UK
9. London International Development Centre (LIDC), London, UK

Authors' emails

BMK: benjamin.kadia@lstmed.ac.uk; ASR: anouschka.ramsteijn@abdn.ac.uk; TD: teenarajeev@rediffmail.com; UF: umifahmida@gmail.com; BK: dr.bharatikulkarni@gmail.com; BF: babacar2.faye@ucad.edu.sn; MKH: kyawhtet@gmail.com; DS: doudou.sow@ugb.edu.sn; RRK: rkrajender@yahoo.com, RS: jnb.ritusharma@gmail.com, ARPS: arientasudibya@gmail.com; SK: skusuma@seameo-recfon.org; TCA: tiffany.angelin@gmail.com; MN: mifanurfadilah@gmail.com; MLJ: modou.jobarteh@lshtm.ac.uk; NSD: yayoumbayediop@gmail.com; IG: igabain19@rvc.ac.uk; BC: bcalvo@rvc.ac.uk; EF: elaine.ferguson@lshtm.ac.uk; PH:paul.haggarty@abdn.ac.uk; CH: Claire.Heffernan@lidc.ac.uk; JPW: jowebster@rvc.ac.uk; AWW: alan.walker@abdn.ac.uk; SJA: stephen.allen@lstmed.ac.uk;

Corresponding author: Benjamin Momo Kadia: Department of Clinical Sciences, LSTM; email: benjamin.kadia@lstmed.ac.uk

Confidential: For Review Only

ABSTRACT

Introduction: Childhood stunting has a complex aetiology, with poor gut health being an important contributor. This study will assess inter-relationships between gut health indices including bacterial and parasitic colonisation of the gut, the gut microbiota, and biomarkers of environmental enteric dysfunction (EED), and linear growth, systemic inflammation, and growth hormones in early childhood.

Methods and analysis: A longitudinal study of observation cohorts of 600 newborns and their mothers in India, Indonesia, and Senegal will be conducted. Women will be recruited during pregnancy and their children followed up to age 24 months. Stool, urine, and blood samples will be collected from the women and children for assessments of helminthic and protozoal parasites, bacterial pathogens, faecal microbiota taxa, biomarkers of EED, systemic inflammation, and growth hormones. Child anthropometric measurements will be collected at birth and at ages 3, 6, 9, 12, 18 and 24 months. The gut health indices will be integrated with cohort data from other Action Against Stunting Hub (AASH) workstreams for interdisciplinary analyses of childhood stunting and the development of a new typology of stunting.

Discussion: This study will advance scientific understanding of the role of gut health in childhood stunting and will contribute to a broader understanding of the complex aetiologies of this condition as part of the interdisciplinary research of the AASH to reduce the global burden of childhood stunting.

Ethics and dissemination: This study has been approved by the relevant Ethics Committees in Senegal, India, and Indonesia and LSHTM. The results will be submitted for publication in peer-reviewed journals.

Confidential: For Review Only

1 **SUMMARY BOX**

What is known about the subject?

- In areas with poor hygiene and sanitation, recurrent enteric infection and alterations in the gut microbiota are associated with environmental enteric dysfunction (EED).
- EED is an important precursor of childhood stunting.

What this study hopes to add

- To compare characteristics of gut health in mothers and their infants and elucidate their association with childhood stunting across three distinct geographical settings, with the inclusion of mothers being a novel approach to provide more robust evidence on early life determinants of childhood stunting.
- To advance the scientific understanding of the role of gut health in childhood stunting.
- To contribute to the development of a new typology of childhood stunting based on knowledge of the relationship between the gut and other host and environmental factors, and to support development of interdisciplinary approaches to prevent and reduce childhood stunting.

2
3
4

Confidential: For Review Only

Introduction

More than 1 in 5 (about 149.2 million worldwide) children under-five years of age are currently stunted [1]. Childhood stunting is defined as having a height-for-age z-score more than 2 standard deviations below the median of the WHO child growth standards [2]. Whilst the aetiology of stunting is complex, several studies suggest that it can be associated with environmental enteric dysfunction (EED) resulting from asymptomatic gut infection with pathogenic microbes [3,4]. EED refers to the abnormal gut structure and function commonly found in people living in environments with poor sanitation and hygiene, with onset detected as early as 12 weeks after birth despite exclusive breast feeding [5,6].

EED is characterised by villous atrophy, crypt hyperplasia, chronic enteric inflammation, and increased gut mucosal permeability. It leads to reduced nutrient absorption, increased risk of systemic infection due to reduced integrity of the gut barrier, particularly due to gut-derived Gram-negative bacteria, and impaired immune responses to oral vaccines [5,6]. EED contributes to stunting through nutrient malabsorption and systemic inflammation, with reduced production of growth hormones [5–7]. Furthermore, EED has been associated with disruption in the ecological niches that support the gut microbiota [5]. It is known that malnutrition and growth faltering in early life are associated with microbiota immaturity [8], and experimental evidence suggests that the gut microbiota may be causally involved in the manifestation of stunting [9]. The gut microbiota also plays an important role in protecting against colonisation and invasion of the gut by pathogens [6].

Previous studies of stunting have explored various biomarkers of EED including plasma α_1-acid glycoprotein (AGP), which is a longer-term marker of systemic inflammation [7,10–15], faecal myeloperoxidase (MPO, a marker of intestinal inflammation) [11–13,16], faecal α_1-antitrypsin (AAT, a marker of protein loss and intestinal permeability) [11,13,16] and plasma intestinal fatty acid binding protein (IFABP; mucosal permeability/gut integrity) [7,10,16,17] and also measured plasma growth hormones (insulin-like growth factor (IGF)-1 and its binding protein IGFBP3) [7,10,12]. However, a consensus has not been reached on the biomarkers to use as standard for diagnosis and monitoring of EED. Thus, in low-income settings where EED and stunting are most prevalent, the association of biomarkers of EED with linear growth requires further investigation.

In this study, we will assess aspects of gut health, including gut microbiota profiles, colonisation of the gut by pathogenic microbes (bacterial and parasitic pathogens), and biomarkers of EED and associated pathology (systemic inflammation and reduced growth hormones) during pregnancy and early childhood, and their role in childhood stunting.

https://mc.manuscriptcentral.com/bmjpo

Confidential: For Review Only

METHODS AND ANALYSIS

Study design and population

This study is a longitudinal, observational study led by the UKRI GCRF Action Against Stunting Hub (AASH). Women in the second and third trimesters of pregnancy will be recruited in Kaffrine District in Senegal (rural), Hyderabad in India (urban), and East Lombok in Indonesia (rural), and their newborns will be followed up to age 24 months.

Study procedures

Sample collection

Stool samples will be collected from pregnant women in the third trimester. In infants/children, stool samples will be collected either at a health facility or in the participants' homes at ages 1 month, 6 months and 24 months. For stool samples collected at home, study staff will visit either on the day before or on the morning of sample collection to provide the mother/carer with a disposable nappy or plastic sheet, sample collection containers with spatula, plastic specimen bag, small cold box with ice pack and a bar of soap. Mothers/carers will be asked to snugly fit a nappy or plastic sheet to the infant last thing at night and collect the next stool produced by the infant either by pouring it from the nappy/sheet, if the sample is loose, or using the spatula if formed and fill the sample containers. They will place the filled container in the specimen bag and then in the cold box and wash their hands with soap. Field staff will return later the same day or the following day to label the sample and transfer it to the laboratory. Subject to agreement with the mother/carer, this process will be repeated over the following days until an adequate stool sample is obtained (at least 5 ml of liquid stool or 5 grams of formed stool). To avoid dilution of biomarkers in children suffering from acute diarrhoea, stool collection will be delayed until the stools have returned to their usual consistency. Acute diarrhoea will be considered as significantly more frequent or loose/watery stools in the mother's/carer's opinion in exclusively breastfed babies and three or more liquid stools in the last 24 hours in children receiving other feeds.

During the visits to collect samples in Indonesia and India at 6 months and in Senegal at age 6 and 24 months, trained research staff will collect 2.5 ml venous blood. Subject to the agreement of the mother/carer, no more than three attempts will be performed to collect the required blood volume.

Finally, early morning urine samples will be collected from pregnant mothers and infants at 6 and 24 months in Senegal to identify the presence and intensity of *Schistosoma haematobium* and hybrids therein.

Confidential: For Review Only

All samples will be transported to laboratories in a cold box. In Indonesia, blood samples will be wrapped in plastic and transported in a car freezer. The time between sample collection and receipt in the laboratory will be recorded.

Anthropometric measurements (including length and weight) will be taken in duplicate from infants at birth, 3, 6, 9, 12 18 and 24 months of age as described in detail elsewhere (UKRI GCRF AASH Nutrition workstream protocol paper). Z-scores for length-for-age (LAZ) will be calculated using the 2006 WHO Growth Standards and stunted growth will be defined as a LAZ <-2 standard deviations [2].

Laboratory analysis

Approximately 1 gram of faecal material will be aliquoted into a DNA/RNA Shield Faecal Collection Tube containing a preservative buffer solution (Zymo Research, California, USA) for DNA extraction using the ZymoBIOMICS DNA Miniprep kit (Zymo Research, California, USA) for subsequent microbiome and qPCR-based analyses. Stool samples and aliquots in DNA/RNA Shield will be stored at -20°C until further processing.

Kato-Katz thick smear microscopy (duplicate slides per single stool) will be performed on fresh (untreated) stool to obtain individual egg counts of key helminth parasites reported to be associated with physical and cognitive stunting in children (e.g. *Ascaris* spp., hookworm, *Trichuris trichiura, Hymenolepis nana*). Slides will be read within 60 minutes post Kato-Katz preparation to obtain hookworm data. The slides will then be kept at room temperature prior to microscopic examination of the remaining parasites present. The amount of stool placed on the Kato Katz slide is 41.7 mg, so the numerical values obtained will be multiplied by 24 to give the number of eggs per gram (epg)/Faecal Egg Count (FEC) – the standard measurement to assess the intensity of infection.

Multi-parallel qPCR on DNA extracted from faeces will be performed for high sensitivity and specificity identification of key gastrointestinal parasites [18], based on a modified protocol with updated primers and quality control [19,20]. This is a high throughput species-specific molecular diagnostic tool for routine surveillance, monitoring and research, which allows more accurate identification and quantification of key pathogens associated with physical and cognitive stunting in children relative to slide-based microscopy alone [19,20]. Infection levels can be more precisely measured in low prevalence settings where intensities are low (as predicted in infants). This will target *Ascaris lumbricoides*, *Ancylostoma duodenale*, *Necator americanus*, *Trichuris trichiura*, *Strongyloides stercoralis*, and protozoans such as *Giardia lamblia*, *Cryptosporidium parvum/hominis* and *Entamoeba histolytica/dispar*.

Multiplex PCR will also be used to identify pathogenic strains of *Escherichia coli* (*E. coli*): enteropathogenic *E. coli*, enteroaggregative *E. coli*, enterotoxigenic *E. coli*, and enteroinvasive *E. coli* [21]. In addition, analysis of the faecal microbiota (see below) may also putatively

https://mc.manuscriptcentral.com/bmjpo

Confidential: For Review Only

identify potential enteropathogens at the genus level, such as *Helicobacter, Campylobacter*, *Plesiomonas*, *Yersinia*, *Aeromonas, Shigella/Escherichia,* and *Salmonella* spp..

For microbial culture to detect *Salmonella* spp. and *Shigella* spp., swabbing onto agar plates or inoculating approximately 1 g of fresh stool in Selenite broth for enrichment followed by culture in selective media MacConkey Agar and Xylose Lysine Deoxycholate Agar (MCA and XLD) or by automated culture in Senegal will be done immediately after samples are brought to the laboratory. Gram staining and biochemical tests such as Kligler Iron Agar (KIA), Motility Indole Ornithine (MIO), Lysine decarboxylase (LDC), Urea and Oxidase will be used for identifying the cultured isolates at the genus level, complemented by serological tests to identify the bacterial species using BD Difco Salmonella O Antiserum Poly A-I and BD Difco Shigella Antiserum Poly Group A, A1, B, C, C1, C2 and D.

Myeloperoxidase and alpha 1-antitrypsin will be measured in stool samples using the Immunodiagnostik AG (Bensheim, Germany) ELISA Kit. Sample preparation and analysis will be as per manufacturer's instructions.

Microbial profiling will be done by 16S rRNA gene amplicon sequencing. We will use barcoded primers from the Earth Microbiome Project protocol [22], with slight modifications to increase detected diversity [23], to amplify the V4 region of the 16S rRNA gene using PCR on DNA extracted from stool samples. 16S rRNA gene amplicon sequencing will be carried out using the Illumina MiSeq platform. For amplicons from Indonesia and Senegal this will be carried out by sequencing providers in the UK, while sequencing of Indian sample amplicons will take place at a sequencing provider in India. Negative (no DNA template "blanks") and positive (extracted DNA and 16S rRNA gene amplicons from one benchmark stool sample) control samples will be included in each sequencing run for quality control purposes, and to ensure consistency between runs, both longitudinally, and between participant countries.

In the urine samples in Senegal, assessment for blood in urine will be performed immediately upon receipt in the laboratory (Hemastix™ Reagent Strip; Bayer Corp, Diagnostics Div., New York, USA). Standard urine filtration and microscopy (duplicate 10ml per urine sample) will be performed to identify and quantify *Schistosoma haematobium* (and hybrids therein) infection status and recorded as eggs per 10 ml/urine. Positive filters will be placed in fresh water and exposed to light to facilitate egg-hatching into miracidia. Free-swimming miracidia will be individually pipetted onto Whatman Indicating FTA Classic cards (GE Healthcare Life Sciences, UK) for DNA storage and subsequent molecular analysis. Individual *Schistosoma* DNA extracts will be characterised by amplification of a partial fragment of the mitochondrial cytochrome c oxidase subunit 1 (*cox1*) and the complete nuclear ribosomal DNA internal transcribed spacer (ITS) to identify species/species-combination, following protocols described elsewhere [24].

Confidential: For Review Only

Blood samples will be centrifuged and separated on receipt at the laboratory and plasma stored at -80°C until analyses. Samples in Senegal will be stored at -20°C and transported to Dakar within days of collection to store at -80°C. Blood samples will be analysed for biomarkers of inflammation (AGP; Human AGP Quantikine ELISA Kit) and C Reactive Protein (CRP; Human CRP/CRP Quantikine ELISA kit in India and Senegal; Quansys multiplex in Indonesia); gut mucosal integrity (IFABP; Human FABP2/I-FABP Quantikine ELISA Kit in India and Senegal; Quansys multiplex in Indonesia) and plasma growth hormones (IGF-1 and IGF BP3; Human IGF-1 and IGFBP-3 Quantikine ELISA Kits respectively, R&D Systems, Inc. Minneapolis, USA in India and Senegal and Quansys multiplex in Indonesia). Assays will be carried out according to the manufacturers' instructions at the National Institute of Nutrition, Hyderabad in India and the Université Cheikh Anta DIOP, Dakar in Senegal. In Indonesia, sample preparation will be done in Lombok and analyses in SEAMEO RECFON's laboratory in Jakarta.

All participants with clinically relevant bacterial or parasitic infections will be offered treatment. After these analyses, sample aliquots will be stored at -80°C.

Sample size determination

The MAL-ED birth cohort study found a statistically significant association between raised plasma AGP and decreased linear growth in 1476 plasma samples from children aged 4-11 months from eight countries [13]. Therefore, it is expected that the current longitudinal study including 600 children in each of the 3 countries will be sufficiently powered to detect a significant association (if any) between AGP and linear growth in blood samples collected at age 6 months.

Data management and analysis

Data management will follow procedures established by the AASH (see the overview of study design, data collection and management procedures of the AASH). This includes a data sharing strategy that will build upon the guiding principles for scientific management and stewardship and Concordat on Open Research Data to ensure equitable access to data.

For microbiota analysis, FASTQ files containing 16S rRNA gene amplicon sequences will be processed using well validated software pipelines such as mothur [25] or DADA2 [26]. These pipelines include various quality control steps and will produce final output tables containing the proportional abundances of various microbiota taxa in each sample. They can also generate various metrics of bacterial diversity within and between samples. These data will be visualised in the form of dendrograms, ordination plots, heatmaps and box plots. Microbiota 16S rRNA gene sequence data will be released publicly via the European Nucleotide Archive at the end of the study.

Confidential: For Review Only

Multi-level models taking into consideration aggregate units such as geographical setting, will be used to investigate associations between enteropathogens/gut parasites, biomarker concentrations and LAZ, to assess longitudinal associations between biomarker concentrations and growth rates, and to determine which biomarkers, enteropathogens and/or characteristics of the gut microbiota are associated with stunted growth at specific timepoints. In addition, Bayesian methods, using Directed Acyclic Graphs, will be used to investigate a variety of possible interactions between gut health indices and linear growth (stunting) and to assess combinatorial effects of the diverse elements of gut health (expressed as categorical or continuous variables, as appropriate) on stunting.

To allow for interdisciplinary analyses and the development of the typology of stunting, multi-level modelling and Bayesian methods will be extended to integrate cohort data collected in other workstreams. Stata version 17 and other appropriate software will be used for the analysis.

Patient and public involvement

There was no involvement of patients or the public in the design of this study.

Confidential: For Review Only

Discussion

The limited impact of interventions to prevent or ameliorate stunting in young children in low-resource settings has resulted in a renewed interest in the role of gut health. The AASH cohorts will be amongst the largest investigating the inter-relationships between gut colonisation with bacterial, protozoal and helminth pathogens, the gut microbiota, and biomarkers of EED on linear growth in populations with different prevalence rates of stunting (moderate to high) and environmental conditions.

The AASH offers the opportunity to compare gut health characteristics and their association with stunting across three distinct geographical settings. This is particularly important as there is limited evidence to date regarding the role of gut health in children in West Africa and Indonesia. In addition, our findings will be integrated with data collected in other AASH workstreams to advance scientific understanding of how gut health interacts with other host and environmental factors that underlie common typologies of stunting and increase the evidence base for future intervention studies. Harmonised procedures to collect, manage and analyse data and samples across the countries will greatly enhance the validity of our findings.

In low-income settings, there is a need to further investigate biomarkers of intestinal structure and function to identify acceptable and affordable tools that can be scaled-up at the population level to diagnose and monitor EED and identify possible geographic-specific and seasonal variations in the association between these biomarkers and stunting. The selection of biomarkers for this study was informed by previous reports of their utility in reflecting the aetiopathogenesis of EED and association with linear growth faltering [10, 12, 13, 15, 16].

This study will have some limitations. Although our study design allows us to explore similarities and differences across cohorts from three countries, our findings may not be generalisable to gut health and growth in other low-resource settings where the intensity of infection, characteristics of the gut microbiota and pathology of EED may differ. In general, it is a challenge to move from associations to an understanding of causality in an observational study. However, the SENeGal SYNbiotic (SENGSYN) study, a clinical trial nested within the AASH investigating the effect of supplementation with synbiotics on growth and biomarkers of gut health (see supplement protocol titled 'Improving gut health and growth in early life: a protocol for an individually randomised, two-arm, 2 open-label, controlled trial of a synbiotic in infants in Kaffrine District, Senegal'), may build on associations between gut health and growth determined in the observation cohorts to provide evidence of causality. From a technical perspective, our method of stool collection using cold boxes and minimising time between sample provision and processing in the laboratories is pragmatic. However, we cannot exclude the possibility that changes in stool biomarkers, pathogens and the microbiota may occur during transfer. As part of quality control, we will assess whether time between collection and receipt in the laboratory is associated with the results of the various analyses.

https://mc.manuscriptcentral.com/bmjpo

Conclusions

The findings of this study will improve scientific understanding of how gut health contributes to stunting, either directly, or through inter-links with the wide range of host and environmental factors monitored in the AASH project, and how these may vary in different geographic locations. Our findings will inform the development of diagnostic and management options for EED and identify biomarkers of gut health that may serve as outcome measures in future intervention trials in resource-constrained settings.

Confidential: For Review Only

Abbreviations

AASH	Action Against Stunting Hub
AGP	α_1-acid glycoprotein
CRP	C-reactive protein
EED	Environmental enteric dysfunction
IFABP	Intestinal fatty acid binding protein
IGF-1	Insulin-like growth factor-1
IGFBP3	Insulin-like growth factor binding protein 3

Confidential: For Review Only

References

1. World Health Organisation (WHO), World Bank, United Nations International Children's Emergency Fund. Levels and trends in child malnutrition: Key findings of the 2020 Edition of the Joint Child Malnutrition Estimates. Available at https://www.who.int/publications-detail-redirect/9789240025257

2. WHO Multicentre Growth Reference Study Group. WHO Child Growth Standards based on length/height, weight and age. Acta Paediatr Suppl. Norway; 2006;450:76–85. doi.10.1111/j.1651-2227.2006.tb02378.x

3. Owino V, Ahmed T, Freemark M, et al. Environmental enteric dysfunction and growth failure/stunting in global child health. Pediatrics. 2016; 38(6): e20160641. doi: 10.1542/peds.2016-0641

4. Nataro JP, Guerrant RL. Chronic consequences on human health induced by microbial pathogens: Growth faltering among children in developing countries. Vaccine. 2017;35:6807–6812. doi.10.1016/j.vaccine.2017.05.035

5. Tickell KD, Atlas HE, Walson JL. Environmental enteric dysfunction: A review of potential mechanisms, consequences and management strategies. BMC Med. 2019;17:1–9. doi.10.1186/s12916-019-1417-3

6. Naylor C, Lu M, Haque R, et al. Environmental Enteropathy, Oral Vaccine Failure and Growth Faltering in Infants in Bangladesh. EBioMedicine. 2015;2:1759–1766. doi.10.1016/j.ebiom.2015.09.036

7. Prendergast AJ, Rukobo S, Chasekwa B, et al. Stunting is characterized by chronic inflammation in zimbabwean infants. PLoS One. 2014;9:e86928. doi.10.1371/journal.pone.0086928

8. Subramanian S, Huq S, Yatsunenko T, et al. Persistent gut microbiota immaturity in malnourished Bangladeshi children. Nature. 2014;510:417–21. doi.10.1038/nature13421

9. Blanton L V, Charbonneau MR, Salih T, et al. Gut bacteria that prevent growth impairments transmitted by microbiota from malnourished children. Science. 2016;351:854. doi. 10.1126/science.aad3311

10. Arndt MB, Cantera JL, Mercer LD, et al. Validation of the Micronutrient and Environmental Enteric Dysfunction Assessment Tool and evaluation of biomarker risk factors for growth faltering and vaccine failure in young Malian children. PLoS Negl Trop Dis. 2020;14:e0008711. doi.10.1371/journal.pntd.0008711

11. Campbell RK, Schulze KJ, Shaikh S, et al. Environmental enteric dysfunction and systemic inflammation predict reduced weight but not length gain in rural Bangladeshi children. Br J Nutr. 2018; ;119(4):407-414. doi: 10.1017/S0007114517003683

12. Iqbal NT, Sadiq K, Syed S, et al. Promising Biomarkers of Environmental Enteric Dysfunction: A Prospective Cohort study in Pakistani Children. Sci Rep. 2018;8:2966. doi.10.1038/s41598-018-21319-8

Confidential: For Review Only

13. Kosek MN, Ahmed T, Bhutta ZA, et al. Causal Pathways from Enteropathogens to Environmental Enteropathy: Findings from the MAL-ED Birth Cohort Study. EBioMedicine. 2017;18:109–117. doi.10.1016/j.ebiom.2017.02.024

14. Merrill RD, Burke RM, Northrop-Clewes CA, et al. Factors associated with inflammation in preschool children and women of reproductive age: Biomarkers Reflecting Inflammation and Nutritional Determinants of Anemia (BRINDA) project. Am. J. Clin. Nutr. 2017. doi.10.3945/ajcn.116.142315

15. Lauer JM, Ghosh S, Ausman LM, et al. Markers of Environmental Enteric Dysfunction Are Associated with Poor Growth and Iron Status in Rural Ugandan Infants. J Nutr. 2020;150:2175–82. doi: 10.1093/jn/nxaa141

16. Guerrant RL, Leite AM, Pinkerton R, Medeiros PHQS, Cavalcante PA, DeBoer M, et al. Biomarkers of environmental enteropathy, inflammation, stunting, and impaired growth in children in northeast Brazil. PLoS One. 2016;11:e0158772. doi.10.1371/journal.pone.0158772

17. Zambruni M, Ochoa TJ, Somasunderam A, et al. Stunting Is Preceded by Intestinal Mucosal Damage and Microbiome Changes and Is Associated with Systemic Inflammation in a Cohort of Peruvian Infants. Am J Trop Med Hyg. 2019;101:1009–17. doi.10.4269/ajtmh.18-0975

18. Easton A V, Oliveira RG, O'Connell EM, et al. Multi-parallel qPCR provides increased sensitivity and diagnostic breadth for gastrointestinal parasites of humans: field-based inferences on the impact of mass deworming. Parasit Vectors. 2016;9:38. doi.10.1186/s13071-016-1314-y

19. Mejia R, Vicuña Y, Broncano N, et al. A novel, multi-parallel, real-time polymerase chain reaction approach for eight gastrointestinal parasites provides improved diagnostic capabilities to resource-limited at-risk populations. Am J Trop Med Hyg. 2013/03/18. The American Society of Tropical Medicine and Hygiene; 2013;88:1041–7. doi.10.4269/ajtmh.12-0726

20. Papaiakovou M, Gasser RB, Littlewood DTJ. Quantitative PCR-Based Diagnosis of Soil-Transmitted Helminth Infections: Faecal or Fickle? Trends Parasitol. 2019;35:491–500. doi.10.1016/j.pt.2019.04.006

21. Zhang J, Xu Y, Ling X, et al. Identification of diarrheagenic Escherichia coli by a new multiplex PCR assay and capillary electrophoresis. Mol Cell Probes. 2020;49:101477. doi.10.1016/j.mcp.2019.101477

22. Caporaso JG, Lauber CL, Walters WA, et al. Global patterns of 16S rRNA diversity at a depth of millions of sequences per sample. Proc Natl Acad Sci U S A. 2011;108 Suppl:4516–22. doi.10.1073/pnas.1000080107

23. Walters W, Hyde ER, Berg-Lyons D, et al. Improved Bacterial 16S rRNA Gene (V4 and V4-5) and Fungal Internal Transcribed Spacer Marker Gene Primers for Microbial Community Surveys. mSystems. 2016;1:e00009-15. doi.10.1128/mSystems.00009-15

24. Léger E, Borlase A, Fall CB, et al. Prevalence and distribution of schistosomiasis in human, livestock, and snail populations in northern Senegal: a One Health epidemiological study of a multi-host system. Lancet Planet Heal. 2020;4:e330–42. doi.10.1016/S2542-5196(20)30129-7

Confidential: For Review Only

25. Schloss PD, Westcott SL, Ryabin T, et al. Introducing mothur: open-source, platform-independent, community-supported software for describing and comparing microbial communities. Appl Environ Microbiol. 2009;75:7537–41. doi.10.1128/AEM.01541-09

26. Callahan BJ, McMurdie PJ, Rosen MJ, et al. DADA2: High-resolution sample inference from Illumina amplicon data. Nat Methods. 2016;13:581–3. doi.10.1038/nmeth.3869

Confidential: For Review Only

Footnotes

Contributors

BMK: Clinical Research Associate dedicated to the overview and delivery of the Gut Health workstream of the AASH; drafted the initial manuscript.
ASR: Provides research and organisational support in the microbiome component of the study; critically revised the manuscript.
TD: Theme lead for gut enteropathogens and parasitology, India; lead for the development of standard operating procedures.
UF: Country lead, AASH, Indonesia; contributed to study design and coordination of the study and development of standard operating procedures.
BK: Country lead, AASH, India; contributed to the design and coordination of the study in India.
BF: Country lead, AASH, Indonesia, and contributed to the design of the study and coordinates the study in Senegal.
MKH: Gut Health theme lead, Indonesia; contributes to the implementation of the study in Indonesia.
DS: Gut Health theme lead, Senegal; oversees the implementation of the study in Senegal.
RRK: supports coordination of laboratory analysis in India; reviewed the manuscript
RS: field work support and data collection in India; reviewed the manuscript
ARPS, SK, TCA and MN: Actively contribute to the set-up and implementation of the AASH in Indonesia.
MLJ: Edited the manuscript; reviewed the manuscript for technical and intellectual consistency
NSD: Participates in the set-up and implementation of the study in Senegal.
IG: Provides research and organisational support in the parasitology aspect of the study
BC: Provided guidance on preparationof data management and statistical analysis plan for the Gut Health workstream
EF: Oversees the Nutrition/Anthropometry workstream of the Stunting Hub; contributed to the design of the study, and critically revised the protocol.
PH and CH: Contributed to the conception of the study and critically revised the protocol
JPW: Conception of the study; oversees the parasitology theme of the study; critically revised the protocol
AWW: Conception of the study, oversees the microbiome component of the study; critically revised the protocol and supervised drafting of the manuscript.
SJA: Conception of the study; oversees the delivery of the Gut Health workstream; critically revised the protocol and supervised drafting of the manuscript.

Funding: This work is supported by the London School of Hygiene and Tropical Medicine through funding from the UKRI GCRF Action Against Stunting Hub. AWW and PH are supported

Confidential: For Review Only

by core funding from the Scottish Government's Rural and Environment Science and Analytical Services (RESAS) division.

Competing interests: The authors declare that they have no competing interests.

Consent for publication: Not required.

Ethical considerations: This study has been approved by the ethics committees of the National Institute for Nutrition, India and the University of Indonesia, the National Ethics Committee for Scientific Research in Senegal, and the Research Ethics Committee of the London School of Hygiene and Tropical Medicine in the UK.

Data availability statement: No data are available. Data sharing is not applicable to this manuscript as it describes a protocol.

Confidential: For Review Only

BMJ Paediatrics Open

Assessment of the role of gut health in childhood stunting in a multi-site, longitudinal study in India, Indonesia, and Senegal: a UKRI GCRF Action Against Stunting Hub protocol

Journal:	*BMJ Paediatrics Open*
Manuscript ID	bmjpo-2022-001637.R1
Article Type:	Protocol
Date Submitted by the Author:	02-Nov-2022
Complete List of Authors:	Momo Kadia, Benjamin; Liverpool School of Tropical Medicine Department of Clinical Sciences<br>Ramsteijn, Anouschka; University of Aberdeen<br>Dasi, Teena; National Institute of Nutrition<br>Fahmida, Umi; SEAMEO Regional Center for Food and Nutrition<br>Kulkarni, Bharati; National Institute of Nutrition<br>Faye, Babacar; Cheikh Anta Diop University of Dakar<br>Htet, Min Kyaw; SEAMEO Regional Center for Food and Nutrition<br>Sow, Doudou; Université Gaston Berger<br>Kalashikam, Rajender Rao; National Institute of Nutrition<br>Sharma, Ritu; National Institute of Nutrition<br>Sudibya, Arienta R.P.; SEAMEO Regional Center for Food and Nutrition<br>Kusuma, Sari; SEAMEO Regional Center for Food and Nutrition<br>Angelin, Tiffany C.; SEAMEO Regional Center for Food and Nutrition<br>Nurfadilah, Mifa; SEAMEO Regional Center for Food and Nutrition<br>Jobarteh, Modou; London School of Hygiene & Tropical Medicine<br>Diop, Ndeye Sokhna; Cheikh Anta Diop University of Dakar<br>Gabain, Isobel; University of London, Department of Pathobiology and Population Sciences<br>Calvo-Urbano, Beatriz; University of London, Department of Pathobiology and Population Sciences<br>Ferguson, Elaine; London School of Hygiene & Tropical Medicine<br>Haggarty, Paul; University of Aberdeen<br>Heffernan, Claire; London School of Hygiene & Tropical Medicine; London International Development Centre<br>Webster, Joanne P.; University of London, Department of Pathobiology and Population Sciences<br>Walker, Alan; University of Aberdeen<br>Allen, Stephen; Liverpool School of Tropical Medicine Department of Clinical Sciences
Keywords:	Gastroenterology, Growth, Microbiology, Molecular Biology, Parasitology

I, the Submitting Author has the right to grant and does grant on behalf of all authors of the Work (as defined in the below author licence), an exclusive licence and/or a non-exclusive licence for contributions from authors who are: i) UK Crown employees; ii) where BMJ has agreed a CC-BY licence shall apply, and/or iii) in accordance with the terms applicable for US Federal Government officers or employees acting as part of their official duties; on a worldwide, perpetual, irrevocable, royalty-free basis to BMJ Publishing Group Ltd ("BMJ") its licensees and where the relevant Journal is co-owned by BMJ to the co-owners of the Journal, to publish the Work in this journal and any other BMJ products and to exploit all rights, as set out in our licence.

The Submitting Author accepts and understands that any supply made under these terms is made by BMJ to the Submitting Author unless you are acting as an employee on behalf of your employer or a postgraduate student of an affiliated institution which is paying any applicable article publishing charge ("APC") for Open Access articles. Where the Submitting Author wishes to make the Work available on an Open Access basis (and intends to pay the relevant APC), the terms of reuse of such Open Access shall be governed by a Creative Commons licence – details of these licences and which Creative Commons licence will apply to this Work are set out in our licence referred to above.

Other than as permitted in any relevant BMJ Author's Self Archiving Policies, I confirm this Work has not been accepted for publication elsewhere, is not being considered for publication elsewhere and does not duplicate material already published. I confirm all authors consent to publication of this Work and authorise the granting of this licence.

Confidential: For Review Only

Assessment of the role of gut health in childhood stunting in a multi-site, longitudinal study in India, Indonesia, and Senegal: a UKRI GCRF Action Against Stunting Hub protocol

Benjamin Momo Kadia[1], Anouschka S. Ramsteijn[2], Teena Dasi[3], Umi Fahmida[4], Bharati Kulkarni[3], Babacar Faye[5], Min Kyaw Htet[4], Doudou Sow[6], Rajender Rao Kalashikam[3], Ritu Sharma[3], Arienta R.P. Sudibya[4], Sari Kusuma[4], Tiffany C. Angelin[4], Mifa Nurfadilah[4], Modou L. Jobarteh[7], Ndeye Sokhna Diop[5], Isobel Gabain[8], Beatriz Calvo-Urbano[8], Elaine Ferguson[7], Paul Haggarty[2], Claire Heffernan[7,8,9], Joanne P. Webster[8 α], Alan W. Walker[2α], Stephen J Allen[1α]

[α]Contributed equally

Authors' affiliations

1. Department of Clinical Sciences, Liverpool School of Tropical Medicine (LSTM), Liverpool, United Kingdom (UK)
2. Rowett Institute, University of Aberdeen, Aberdeen, UK
3. ICMR-National Institute of Nutrition, Hyderabad, India
4. Southeast Asian Ministry of Education Organisation Regional Centre for Food and Nutrition (SEAMEO RECFON), East Jakarta, Indonesia
5. Service de Parasitologie-Mycologie, Faculté de Médecine, Université Cheikh Anta Diop (UCAD), Dakar, Sénégal
6. Service de Parasitologie-Mycologie, UFR Sciences de la Santé, Université Gaston Berger, Saint Louis, Sénégal
7. London School of Hygiene and Tropical Medicine (LSHTM), London, UK
8. Department of Pathobiology and Population Sciences, Royal Veterinary College, University of London, London, UK
9. London International Development Centre (LIDC), London, UK

Authors' emails

BMK: benjamin.kadia@lstmed.ac.uk; ASR: anouschka.ramsteijn@abdn.ac.uk; TD: teenarajeev@rediffmail.com; UF: umifahmida@gmail.com; BK: dr.bharatikulkarni@gmail.com; BF: babacar2.faye@ucad.edu.sn; MKH: kyawhtet@gmail.com; DS: doudou.sow@ugb.edu.sn; RRK: rkrajender@yahoo.com, RS: jnb.ritusharma@gmail.com, ARPS: arientasudibya@gmail.com; SK: skusuma@seameo-recfon.org; TCA: tiffany.angelin@gmail.com; MN: mifanurfadilah@gmail.com; MLJ: modou.jobarteh@lshtm.ac.uk; NSD: yayoumbayediop@gmail.com; IG: igabain19@rvc.ac.uk; BC: bcalvo@rvc.ac.uk; EF: elaine.ferguson@lshtm.ac.uk; PH:paul.haggarty@abdn.ac.uk; CH: Claire.Heffernan@lidc.ac.uk; JPW: jowebster@rvc.ac.uk; AWW: alan.walker@abdn.ac.uk; SJA: stephen.allen@lstmed.ac.uk;

Corresponding author: Benjamin Momo Kadia: Department of Clinical Sciences, LSTM; email: benjamin.kadia@lstmed.ac.uk

Confidential: For Review Only

ABSTRACT

Introduction: Childhood stunting has a complex aetiology, with poor gut health being an important contributor. This study will assess inter-relationships between maternal and infant gut health indices and infant linear growth. Inter-relationships between gut health indices, systemic inflammation, and growth hormones in early childhood will also be assessed.

Methods and analysis: A longitudinal observational study of cohorts of 600 newborns and their mothers in India, Indonesia, and Senegal will be conducted. Women will be recruited during pregnancy and their children followed up to age 24 months. Stool, urine, and blood samples will be collected from the women and children for assessments of helminthic and protozoal parasites, bacterial pathogens, faecal microbiota taxa, biomarkers of environmental enteric dysfunction, systemic inflammation, and growth hormones. Child anthropometric measurements will be collected at birth and at ages 3, 6, 9, 12, 18 and 24 months. The gut health indices will be integrated with cohort data from other Action Against Stunting Hub (AASH) workstreams for interdisciplinary analyses of childhood stunting and the development of a new typology of stunting.

Discussion: This study will advance scientific understanding of the role of gut health in childhood stunting and will contribute to a broader knowledge of the complex aetiology of this condition as part of the interdisciplinary AASH research to reduce the global burden of childhood stunting.

Ethics and dissemination: This study has been approved by the relevant Ethics Committees in Senegal, India, and Indonesia and LSHTM. The results will be submitted for publication in peer-reviewed journals.

Confidential: For Review Only

1 **SUMMARY BOX**

What is known about the subject?

- In areas with poor hygiene and sanitation, recurrent enteric infections and alterations in the gut microbiota are associated with environmental enteric dysfunction (EED).
- EED is an important precursor of childhood stunting.

What this study hopes to add

- To compare characteristics of gut health in mothers and their infants and elucidate their association with childhood stunting across three distinct geographical settings, with the inclusion of mothers being a novel approach providing more robust evidence on early life determinants of childhood stunting.
- To contribute to the development of a new typology of childhood stunting based on knowledge of the relationship between the gut and other host- and environmental factors, and to support development of interdisciplinary approaches to prevent and reduce childhood stunting.

2
3
4

https://mc.manuscriptcentral.com/bmjpo

Confidential: For Review Only

1 **Abbreviations**
2

AASH	Action Against Stunting Hub
AGP	α_1-acid glycoprotein
CRP	C-reactive protein
EED	Environmental enteric dysfunction
IFABP	Intestinal fatty acid binding protein
IGF-1	Insulin-like growth factor-1
IGFBP3	Insulin-like growth factor binding protein 3
LAZ	Length-for-age z-score

Confidential: For Review Only

Introduction

Worldwide, more than 1 in 5 children under five years of age are currently stunted, with more than half residing in Asia and two out of five in Africa [1]. Childhood stunting is defined as having a height-for-age z-score more than 2 standard deviations below the median of the WHO child growth standards [2]. Whilst the aetiology of stunting is complex, several studies suggest that it is associated with environmental enteric dysfunction (EED) resulting from asymptomatic gut infection with pathogenic microbes [3,4].

EED refers to the abnormal gut structure and function commonly found in people living in environments with poor sanitation and hygiene, with onset detected as early as 12 weeks after birth despite exclusive breast feeding [5,6]. EED is characterised by villous atrophy, crypt hyperplasia, chronic enteric inflammation, and increased gut mucosal permeability. It leads to reduced nutrient absorption, increased risk of systemic infection due to reduced integrity of the gut barrier, particularly due to gut-derived Gram-negative bacteria, and impaired immune responses to oral vaccines [5,6]. EED contributes to stunting through nutrient malabsorption and systemic inflammation, with reduced production of growth hormones [5–7]. Furthermore, EED has been associated with disruption in the ecological niches that support the gut microbiota [5]. It is known that malnutrition and growth faltering in early life are associated with microbiota immaturity [8], and experimental evidence suggests that the gut microbiota may be causally involved in the manifestation of stunting [9]. The gut microbiota also plays an important role in protecting against colonisation and invasion of the gut by pathogens [6].

Previous studies of stunting have explored various biomarkers of EED, including plasma α_1-acid glycoprotein (AGP, a longer-term marker of systemic inflammation) [7,10–15], faecal myeloperoxidase (MPO, a marker of intestinal inflammation) [11–13,16], faecal α_1-antitrypsin (AAT, a marker of protein loss and intestinal permeability) [11,13,16] and plasma intestinal fatty acid binding protein (IFABP, a marker of mucosal permeability and gut integrity) [7,10,16,17], and also measured plasma growth hormones (insulin-like growth factor (IGF)-1 and its binding protein IGFBP3) [7,10,12]. However, a consensus has not been reached on the biomarkers to use as standard for diagnosis and monitoring of EED. Thus, in low-income settings where EED and stunting are most prevalent, the association of biomarkers of EED with linear growth requires further investigation.

In this study, we will assess aspects of gut health, including gut microbiota profiles, colonisation of the gut any beyond by pathogenic microbes (bacterial and parasitic pathogens), and biomarkers of EED and associated pathology (systemic inflammation and reduced growth hormones) during pregnancy and early childhood, and their role in childhood stunting.

Confidential: For Review Only

METHODS AND ANALYSIS

Study design and population

This is a longitudinal, observational study which is part of the UKRI GCRF Action Against Stunting Hub (AASH). Full details of the overall AASH design are available elsewhere (see the overview of study design, data collection and management procedures of the AASH in the supplement), but details relating to the Gut Health component workstream are summarised below.

Women in the second and third trimesters of pregnancy will be recruited in Kaffrine District in Senegal (rural), Hyderabad in India (urban), and East Lombok in Indonesia (rural), and their newborns will be followed up to age 24 months. The prevalence of stunted growth in children under five is 30.9% in India and 31.8% in Indonesia, and despite having a lower prevalence (17.2%), Senegal is still off-track to achieve a 50% reduced prevalence of stunting by 2030 [1].

Sample size determination

Sample size calculations were performed in R with the package “pwr” and were based on a multiple linear regression analysis with 10 predictors, an effect size of 0.035, power of 80%, significance level of 0.05 and drop-out rate of 20%. These calculations resulted in a total of 592 participants per site.

Study procedures

Sample collection

Stool samples will be collected from pregnant women in the third trimester. In infants/children, stool samples will be collected either at a health facility or in the participants’ homes at ages 1 month, 6 months and 24 months. For stool samples collected at home, study staff will visit either on the day before or on the morning of sample collection to provide the mother/carer with a disposable nappy or plastic sheet, sample collection containers with spatula, plastic specimen bag, small cold box with ice pack, and a bar of soap. Mothers/carers will be asked to snugly fit a nappy or plastic sheet to the infant last thing at night and collect the next stool produced by the infant either by pouring it from the nappy/sheet if the sample is loose, or using the spatula if it is formed, and fill the sample containers. They will place the filled container in the specimen bag and then in the cold box and wash their hands with soap. Field staff will return later the same day or the following day to label the sample and transfer it to the laboratory. Subject to agreement with the mother/carer, this process will be repeated over the following fourteen days until an adequate stool sample is obtained (at least 5 ml of liquid stool or 5 grams of formed stool). To avoid dilution of biomarkers in children suffering from acute diarrhoea, stool collection will be delayed until the stools have returned to their usual

Confidential: For Review Only

consistency. Based on the WHO definition[18], acute diarrhoea will be considered as significantly more frequent or loose/watery stools in the mother's/carer's opinion in exclusively breastfed babies and three or more liquid stools in the last 24 hours in children receiving other feeds. Mothers/carers will be fully briefed on recognising acute diarrhoea as distinct from loose stools within exclusively breast-fed babies. During the visits to collect samples at age 6 and 24 months, trained research staff will collect 2.5 ml venous blood. Subject to the agreement of the mother/carer, efforts will be made to limit attempts to collect the required blood volume to three. Finally, early morning urine samples will be collected from pregnant mothers and infants at 6 and 24 months in Senegal to identify the presence and intensity of *Schistosoma haematobium* and hybrids therein. All samples will be transported to laboratories in a cold box. The time between sample collection and receipt in the laboratory will be recorded. Pilot testing of sample collection processes was conducted in each country.

Anthropometric measurements (including length and weight) will be taken in duplicate from infants at birth, 3, 6, 9, 12 18 and 24 months of age as described in detail elsewhere in the supplement (Anthropometric, biochemical, dietary, morbidity and wellbeing assessment in women and children in Indonesia, India and Senegal: a UKRI GCRF Action Against Stunting Hub protocol). Z-scores for length-for-age (LAZ) will be calculated using the 2006 WHO Growth Standards and stunted growth will be defined as a LAZ <-2 standard deviations [2].

Laboratory analysis

Approximately 1 gram of faecal material will be aliquoted into a DNA/RNA Shield Faecal Collection Tube containing a preservative buffer solution (Zymo Research, California, USA) for DNA extraction using the ZymoBIOMICS DNA Miniprep kit (Zymo Research, California, USA) for subsequent microbiome and qPCR-based analyses. Stool samples and aliquots in DNA/RNA Shield will be stored at -20°C until further processing.

Kato-Katz thick smear microscopy (duplicate slides per single stool) will be performed on fresh (untreated) stool to obtain individual egg counts of key helminth parasites reported and/or predicted to be associated with physical and/or cognitive childhood stunting (e.g. *Ascaris* spp., hookworm, *Trichuris trichiura, Hymenolepis nana*) [19]. Slides will be read within 60 minutes post Kato-Katz preparation to obtain hookworm data. The slides will then be maintained at room temperature prior to microscopic examination of the remaining parasites present. The amount of stool placed on the Kato Katz slide is 41.7 mg, so the numerical values obtained will be multiplied by 24 to give the number of eggs per gram (epg)/Faecal Egg Count (FEC) – the standard measurement to assess the intensity of infection.

Multi-parallel qPCR on DNA extracted from faeces will be performed for high sensitivity and specificity identification of key gastrointestinal parasites [20], based on a modified protocol with updated primers and quality control [21,22]. This is a high throughput, species-specific

Confidential: For Review Only

molecular diagnostic tool for routine surveillance, monitoring and research, which allows more accurate identification and quantification of key pathogens associated with physical and cognitive stunting in children relative to slide-based microscopy alone [21,22]. Infection levels can be more precisely measured in low prevalence settings where intensities are low (as predicted in infants). This will target *Ascaris lumbricoides*, *Ancylostoma duodenale*, *Necator americanus*, *Trichuris trichiura*, *Strongyloides stercoralis*, and protozoans such as *Giardia lamblia*, *Cryptosporidium parvum/hominis* and *Entamoeba histolytica/dispar*.

Multiplex PCR will also be used to identify pathogenic strains of *Escherichia coli* (*E. coli*): enteropathogenic *E. coli*, enteroaggregative *E. coli*, enterotoxigenic *E. coli*, and enteroinvasive *E. coli* [23]. In addition, analysis of the faecal microbiota (see below) may also putatively identify potential enteropathogens at the genus level, such as *Helicobacter, Campylobacter*, *Plesiomonas*, *Yersinia*, *Aeromonas, Shigella/Escherichia,* and *Salmonella* spp..

For microbial culture to detect *Salmonella* spp. and *Shigella* spp., swabbing onto agar plates or inoculating approximately 1 g of fresh stool in Selenite broth for enrichment followed by culture in selective media MacConkey Agar and Xylose Lysine Deoxycholate Agar (MCA and XLD) or by automated culture in Senegal will be done immediately after samples are brought to the laboratory. Gram staining and biochemical tests such as Kligler Iron Agar (KIA), Motility Indole Ornithine (MIO), Lysine decarboxylase (LDC), Urea and Oxidase will be used for identifying the cultured isolates at the genus level, complemented by serological tests to identify the bacterial species using BD Difco Salmonella O Antiserum Poly A-I and BD Difco Shigella Antiserum Poly Group A, A1, B, C, C1, C2 and D.

Myeloperoxidase and alpha 1-antitrypsin will be measured in stool samples using the Immunodiagnostik AG (Bensheim, Germany) ELISA Kit. Sample preparation and analysis will be as per manufacturer's instructions.

Microbial profiling will be done by 16S rRNA gene amplicon sequencing. We will use barcoded primers from the Earth Microbiome Project protocol [24], with slight modifications to increase detected diversity [25], to amplify the V4 region of the 16S rRNA gene using PCR on DNA extracted from stool samples. 16S rRNA gene amplicon sequencing will be carried out using the Illumina MiSeq platform. For amplicons from Indonesia and Senegal this will be carried out by sequencing providers in the UK, while sequencing of Indian sample amplicons will take place at a sequencing provider in India. Negative (no DNA template "blanks") and positive (extracted DNA and 16S rRNA gene amplicons from one benchmark stool sample) control samples will be included in each sequencing run for quality control purposes, and to ensure consistency between runs, both longitudinally, and between participant countries.

In the urine samples in Senegal, assessment for blood in urine will be performed immediately upon receipt in the laboratory (Hemastix™ Reagent Strip; Bayer Corp, Diagnostics Div., New York, USA). Standard urine filtration and microscopy (duplicate 10ml per urine sample) will be

https://mc.manuscriptcentral.com/bmjpo
Confidential: For Review Only

performed to identify and quantify *Schistosoma haematobium* (and hybrids therein) infection status and recorded as eggs per 10 ml/urine. Positive filters will be placed in fresh water and exposed to light to facilitate egg-hatching into miracidia. Free-swimming miracidia will be individually pipetted onto Whatman Indicating FTA Classic cards (GE Healthcare Life Sciences, UK) for DNA storage and subsequent molecular analysis. Individual *Schistosoma* DNA extracts will be characterised by amplification of a partial fragment of the mitochondrial cytochrome c oxidase subunit 1 (*cox1*) and the complete nuclear ribosomal DNA internal transcribed spacer (ITS) to identify species/species-combination, following protocols described elsewhere [24].

Blood samples will be centrifuged and separated on receipt at the laboratory and plasma stored at -80°C until analyses. Samples in Senegal will be stored at -20°C and transported to Dakar within days of collection to store at -80°C. Blood samples will be analysed for biomarkers of inflammation (AGP; Human AGP Quantikine ELISA Kit) and C Reactive Protein (CRP; Human CRP/CRP Quantikine ELISA kit in India and Senegal; Quansys multiplex in Indonesia); gut mucosal integrity (IFABP; Human FABP2/I-FABP Quantikine ELISA Kit in India and Senegal; Quansys multiplex in Indonesia) and plasma growth hormones (IGF-1 and IGF BP3; Human IGF-1 and IGFBP-3 Quantikine ELISA Kits respectively, R&D Systems, Inc. Minneapolis, USA in India and Senegal and Quansys multiplex in Indonesia). Assays will be carried out according to the manufacturers' instructions at the National Institute of Nutrition, Hyderabad in India and the Université Cheikh Anta DIOP, Dakar in Senegal. In Indonesia, sample preparation will be done in Lombok and analyses in SEAMEO RECFON's laboratory in Jakarta.

All participants with clinically relevant bacterial or parasitic infections will be offered treatment. After these analyses, sample aliquots will be stored at -80°C.

Data management and analysis

Data management will follow procedures established by the AASH (see the overview of study design, data collection and management procedures of the AASH). This includes a data sharing strategy that will build upon the guiding principles for scientific management and stewardship and Concordat on Open Research Data to ensure equitable access to data.

For microbiota analysis, FASTQ files containing 16S rRNA gene amplicon sequences will be processed using well validated software pipelines such as mothur [27] or DADA2 [28]. These pipelines include various quality control steps and will produce final output tables containing the proportional abundances of various microbiota taxa in each sample. They can also generate various metrics of bacterial diversity within and between samples. These data will be visualised in the form of dendrograms, ordination plots, heatmaps and box plots. Microbiota 16S rRNA gene sequence data will be released publicly via the European Nucleotide Archive at the end of the study.

Confidential: For Review Only

Multi-level models taking into consideration aggregate units such as geographical setting will be used to investigate associations between enteropathogens/gut parasites, biomarker concentrations and LAZ, to assess longitudinal associations between biomarker concentrations and growth rates, and to determine which biomarkers, enteropathogens and/or characteristics of the gut microbiota are associated with stunted growth at specific timepoints. In addition, Bayesian methods, using Directed Acyclic Graphs, will be used to investigate a variety of possible interactions between gut health indices and linear growth (stunting) and to assess combinatorial effects of the diverse elements of gut health (expressed as categorical or continuous variables, as appropriate) on stunting. When the data being analysed include repeated measurements from the same individual, random intercept models will be employed. The number of missing data for each variable of interest will be reported and the distributions of key variables will be tabulated in order to compare individuals with and without complete data. Analyses will be carried out both on datasets where multiple imputation will be implemented, and on datasets that will be restricted to complete cases.

To allow for interdisciplinary analyses and the development of the typology of stunting, multi-level modelling and Bayesian methods will be extended to integrate cohort data collected in other workstreams. Stata version 17 and other appropriate software will be used for the analysis.

Patient and public involvement

There was no involvement of patients or the public in the design of this study.

For Review Only

Discussion

The limited impact of interventions to prevent or ameliorate stunting in young children in low-resource settings has resulted in a renewed interest in the role of gut health. The AASH cohorts will be amongst the largest investigating the inter-relationships between gut colonisation with bacterial, protozoal and helminth pathogens, the gut microbiota, and biomarkers of EED, and their impact on linear growth in populations with different prevalence rates of stunting (moderate to high) and environmental conditions.

The AASH offers the opportunity to compare gut health characteristics and their association with stunting across three distinct geographical settings. This is particularly important as there is limited evidence to date regarding the role of gut health in children in West Africa and Indonesia. In addition, our findings will be integrated with data collected in other AASH workstreams to advance scientific understanding of how gut health interacts with other host- and environmental factors that underlie common typologies of stunting and increase the evidence base for future intervention studies. This evidence base will be further strengthened by the investigation of elements of maternal gut health as novel drivers of poor gut health and stunted growth in infants. Harmonised procedures to collect, manage and analyse data and samples across the countries will greatly enhance the validity of our findings.

In low-income settings, there is a need to further investigate biomarkers of intestinal structure and function to identify acceptable and affordable tools that can be scaled-up at the population level to diagnose and monitor EED. Further, it is of interest to identify possible geographic-specific and seasonal variations in the association between these biomarkers and stunting. The selection of biomarkers for this study was informed by previous reports of their utility in reflecting the aetiopathogenesis of EED and association with linear growth faltering [10, 12, 13, 15, 16].

This study will have some limitations. Although our study design allows us to explore similarities and differences across cohorts from three countries, our findings may not be generalisable to gut health and growth in other low-resource settings where the intensity of infection, characteristics of the gut microbiota and pathology of EED may differ. In general, it is a challenge to move from associations to an understanding of causality in an observational study. However, the SENeGal SYNbiotic (SENGSYN) study, a clinical trial nested within the AASH investigating the effect of supplementation with synbiotics on growth and biomarkers of gut health (see supplement protocol entitled 'Improving gut health and growth in early life: a protocol for an individually randomised, two-arm, 2 open-label, controlled trial of a synbiotic in infants in Kaffrine District, Senegal'), may build on associations between gut health and growth determined in the observation cohorts to provide evidence of causality. From a technical perspective, our method of stool collection using cold boxes and minimising time between sample provision and processing in the laboratories is pragmatic. However, we cannot exclude

the possibility that changes in stool biomarkers, pathogens and the microbiota may occur during transfer. As part of quality control, we will assess whether time between collection and receipt in the laboratory is associated with the results of the various analyses. Furthermore, we acknowledge that the WHO Child Growth Standards do not necessarily provide a good statistical fit to each country, but these internationally approved standards are widely accepted and facilitate cross-country comparisons, which is considered particularly important when including the three countries in this study. Previous multi-country studies notably, the MAL-ED study[2], have used the same growth standards.

https://mc.manuscriptcentral.com/bmjpo

Confidential: For Review Only

Conclusions

The findings of this study will improve scientific understanding of how gut health contributes to stunting, either directly, or through inter-links with the wide range of host- and environmental factors (including maternal gut health characteristics) monitored in the AASH project, and how these may vary between different geographic locations. Our findings will inform the development of diagnostic and management options for EED and identify biomarkers of gut health that may serve as outcome measures in future intervention trials in resource-constrained settings.

Confidential: For Review Only

References

1. World Health Organisation (WHO), World Bank, United Nations International Children's Emergency Fund. Levels and trends in child malnutrition: Key findings of the 2021 Edition of the Joint Child Malnutrition Estimates. Available at https://www.who.int/publications-detail-redirect/9789240025257

2. WHO Multicentre Growth Reference Study Group. WHO Child Growth Standards based on length/height, weight and age. Acta Paediatr Suppl. Norway; 2006;450:76–85. doi.10.1111/j.1651-2227.2006.tb02378.x

3. Owino V, Ahmed T, Freemark M, et al. Environmental enteric dysfunction and growth failure/stunting in global child health. Pediatrics. 2016; 38(6): e20160641. doi: 10.1542/peds.2016-0641

4. Nataro JP, Guerrant RL. Chronic consequences on human health induced by microbial pathogens: Growth faltering among children in developing countries. Vaccine. 2017;35:6807–6812. doi.10.1016/j.vaccine.2017.05.035

5. Tickell KD, Atlas HE, Walson JL. Environmental enteric dysfunction: A review of potential mechanisms, consequences and management strategies. BMC Med. 2019;17:1–9. doi.10.1186/s12916-019-1417-3

6. Naylor C, Lu M, Haque R, et al. Environmental Enteropathy, Oral Vaccine Failure and Growth Faltering in Infants in Bangladesh. EBioMedicine. 2015;2:1759–1766. doi.10.1016/j.ebiom.2015.09.036

7. Prendergast AJ, Rukobo S, Chasekwa B, et al. Stunting is characterized by chronic inflammation in zimbabwean infants. PLoS One. 2014;9:e86928. doi.10.1371/journal.pone.0086928

8. Subramanian S, Huq S, Yatsunenko T, et al. Persistent gut microbiota immaturity in malnourished Bangladeshi children. Nature. 2014;510:417–21. doi.10.1038/nature13421

9. Blanton L V, Charbonneau MR, Salih T, et al. Gut bacteria that prevent growth impairments transmitted by microbiota from malnourished children. Science. 2016;351:854. doi. 10.1126/science.aad3311

10. Arndt MB, Cantera JL, Mercer LD, et al. Validation of the Micronutrient and Environmental Enteric Dysfunction Assessment Tool and evaluation of biomarker risk factors for growth faltering and vaccine failure in young Malian children. PLoS Negl Trop Dis. 2020;14:e0008711. doi.10.1371/journal.pntd.0008711

11. Campbell RK, Schulze KJ, Shaikh S, et al. Environmental enteric dysfunction and systemic inflammation predict reduced weight but not length gain in rural Bangladeshi children. Br J Nutr. 2018; ;119(4):407-414. doi: 10.1017/S0007114517003683

12. Iqbal NT, Sadiq K, Syed S, et al. Promising Biomarkers of Environmental Enteric Dysfunction: A Prospective Cohort study in Pakistani Children. Sci Rep. 2018;8:2966. doi.10.1038/s41598-018-21319-8

Confidential: For Review Only

13. Kosek MN, Ahmed T, Bhutta ZA, et al. Causal Pathways from Enteropathogens to Environmental Enteropathy: Findings from the MAL-ED Birth Cohort Study. EBioMedicine. 2017;18:109–117. doi.10.1016/j.ebiom.2017.02.024

14. Merrill RD, Burke RM, Northrop-Clewes CA, et al. Factors associated with inflammation in preschool children and women of reproductive age: Biomarkers Reflecting Inflammation and Nutritional Determinants of Anemia (BRINDA) project. Am. J. Clin. Nutr. 2017. doi.10.3945/ajcn.116.142315

15. Lauer JM, Ghosh S, Ausman LM, et al. Markers of Environmental Enteric Dysfunction Are Associated with Poor Growth and Iron Status in Rural Ugandan Infants. J Nutr. 2020;150:2175–82. doi: 10.1093/jn/nxaa141

16. Guerrant RL, Leite AM, Pinkerton R, Medeiros PHQS, Cavalcante PA, DeBoer M, et al. Biomarkers of environmental enteropathy, inflammation, stunting, and impaired growth in children in northeast Brazil. PLoS One. 2016;11:e0158772. doi.10.1371/journal.pone.0158772

17. Zambruni M, Ochoa TJ, Somasunderam A, et al. Stunting Is Preceded by Intestinal Mucosal Damage and Microbiome Changes and Is Associated with Systemic Inflammation in a Cohort of Peruvian Infants. Am J Trop Med Hyg. 2019;101:1009–17. doi.10.4269/ajtmh.18-0975

18. World Health Organisation (WHO). WHO Fact sheets: Diarrhoeal disease (updated 2 May 2017). Available at https://www.who.int/news-room/fact-sheets/detail/diarrhoeal-disease

19. Raj, E., Calvo-Urbano, B., Heffernan, C. *et al.* Systematic review to evaluate a potential association between helminth infection and physical stunting in children. *Parasites Vectors*. 2022; 15, 135. doi.org/10.1186/s13071-022-05235-5

20. Easton A V, Oliveira RG, O'Connell EM, et al. Multi-parallel qPCR provides increased sensitivity and diagnostic breadth for gastrointestinal parasites of humans: field-based inferences on the impact of mass deworming. Parasit Vectors. 2016;9:38. doi.10.1186/s13071-016-1314-y

21. Mejia R, Vicuña Y, Broncano N, et al. A novel, multi-parallel, real-time polymerase chain reaction approach for eight gastrointestinal parasites provides improved diagnostic capabilities to resource-limited at-risk populations. Am J Trop Med Hyg. 2013/03/18. The American Society of Tropical Medicine and Hygiene; 2013;88:1041–7. doi.10.4269/ajtmh.12-0726

22. Papaiakovou M, Gasser RB, Littlewood DTJ. Quantitative PCR-Based Diagnosis of Soil-Transmitted Helminth Infections: Faecal or Fickle? Trends Parasitol. 2019;35:491–500. doi.10.1016/j.pt.2019.04.006

23. Zhang J, Xu Y, Ling X, et al. Identification of diarrheagenic Escherichia coli by a new multiplex PCR assay and capillary electrophoresis. Mol Cell Probes. 2020;49:101477. doi.10.1016/j.mcp.2019.101477

24. Caporaso JG, Lauber CL, Walters WA, et al. Global patterns of 16S rRNA diversity at a depth of millions of sequences per sample. Proc Natl Acad Sci U S A. 2011;108 Suppl:4516–22. doi.10.1073/pnas.1000080107

Confidential: For Review Only

25. Walters W, Hyde ER, Berg-Lyons D, et al. Improved Bacterial 16S rRNA Gene (V4 and V4-5) and Fungal Internal Transcribed Spacer Marker Gene Primers for Microbial Community Surveys. mSystems. 2016;1:e00009-15. doi.10.1128/mSystems.00009-15

26. Léger E, Borlase A, Fall CB, et al. Prevalence and distribution of schistosomiasis in human, livestock, and snail populations in northern Senegal: a One Health epidemiological study of a multi-host system. Lancet Planet Heal. 2020;4:e330–42. doi.10.1016/S2542-5196(20)30129-7

27. Schloss PD, Westcott SL, Ryabin T, et al. Introducing mothur: open-source, platform-independent, community-supported software for describing and comparing microbial communities. Appl Environ Microbiol. 2009;75:7537–41. doi.10.1128/AEM.01541-09

28. Callahan BJ, McMurdie PJ, Rosen MJ, et al. DADA2: High-resolution sample inference from Illumina amplicon data. Nat Methods. 2016;13:581–3. doi.10.1038/nmeth.3869

Confidential: For Review Only

Footnotes

Contributors

BMK: Clinical Research Associate dedicated to the overview and delivery of the Gut Health workstream of the AASH; drafted the initial manuscript.
ASR: Provides research and organisational support in the microbiome component of the study; critically revised the manuscript.
TD: Theme lead for gut enteropathogens and parasitology, India; lead for the development of standard operating procedures.
UF: Country lead, AASH, Indonesia; contributed to study design and coordination of the study and development of standard operating procedures.
BK: Country lead, AASH, India; contributed to the design and coordination of the study in India.
BF: Country lead, AASH, Indonesia, and contributed to the design of the study and coordinates the study in Senegal.
MKH: Gut Health theme lead, Indonesia; contributes to the implementation of the study in Indonesia.
DS: Gut Health theme lead, Senegal; oversees the implementation of the study in Senegal.
RRK: supports coordination of laboratory analysis in India; reviewed the manuscript
RS: field work support and data collection in India; reviewed the manuscript
ARPS, SK, TCA and MN: Actively contribute to the set-up and implementation of the AASH in Indonesia.
MLJ: Edited the manuscript; reviewed the manuscript for technical and intellectual consistency
NSD: Participates in the set-up and implementation of the study in Senegal.
IG: Provided research and organisational support in the parasitology aspect of the study
BC: Provided guidance on preparationof data management and statistical analysis plan for the Gut Health workstream
EF: Oversees the Nutrition/Anthropometry workstream of the Stunting Hub; contributed to the design of the study, and critically revised the protocol.
PH and CH: Contributed to the conception of the study and critically revised the protocol
JPW: Conception of the study; oversees the parasitology theme of the study; critically revised the protocol and manuscript.
AWW: Conception of the study, oversees the microbiome component of the study; critically revised the protocol and supervised drafting of the manuscript.
SJA: Conception of the study; oversees the delivery of the Gut Health workstream; critically revised the protocol and supervised drafting of the manuscript.

Funding: This work is supported by the London School of Hygiene and Tropical Medicine through funding from the UKRI GCRF Action Against Stunting Hub. AWW and PH are supported

Confidential: For Review Only

by core funding from the Scottish Government's Rural and Environment Science and Analytical Services (RESAS) division.

Competing interests: The authors declare that they have no competing interests.

Consent for publication: Not required.

Ethical considerations: This study has been approved by the ethics committees of the National Institute for Nutrition, India and the University of Indonesia, the National Ethics Committee for Scientific Research in Senegal, and the Research Ethics Committee of the London School of Hygiene and Tropical Medicine in the UK.

Data availability statement: No data are available. Data sharing is not applicable to this manuscript as it describes a protocol.

Confidential: For Review Only

LONDON SCHOOL of HYGIENE &TROPICAL MEDICINE

PARTICIPANT CONSENT FORM

Title of Project: UKRI GCRF Action against Stunting Hub – Observational Cohort Study - Mothers
Name of PI/Researcher responsible for project: Professor Claire Heffernan

Statement	Please initial or thumbprint* each box
I confirm that I have read the information sheet dated 26/11/2019 (version 2) for the above named study. I have had the opportunity to consider the information, ask questions and have these answered satisfactorily.<br>**OR**<br>I have had the information explained to me by study personnel in a language that I understand. I have had the opportunity to consider the information, ask questions and have these answered satisfactorily.	
I understand that my participation is voluntary and that I am free to withdraw at any time without giving any reason, without my/my baby's medical care or legal rights being affected.	
I understand that relevant sections of my/my baby's medical notes and data collected during the study may be looked at by authorised individuals from the study team, where it is relevant to my/my baby taking part in this research. I give permission for these individuals to have access to my records. I understand that they will only access information relevant to the birth of my baby.	
I understand that data about me/my baby may be shared via a public data store or by sharing directly with other researchers, and that I/my baby will not be identifiable from this information.	
I understand that the samples of blood, saliva, urine, hair, poo and breastmilk collected from me/my baby may be used to support other research in the future, and may be shared anonymously with other researchers, for their ethically-approved projects.	
I understand and agree that my words may be quoted in publications, reports, web pages, and other research outputs. I understand that I will not be named or in any way personally identifiable in these outputs.	
I understand that I may be contacted at a later date to consider participating in a separate study about my baby's health.	
I agree to take part in the above named study.	

Printed name of participant	Signature of participant	Date

Printed name of impartial witness*	Signature of impartial witness*	Date

I attest that I have explained the study information accurately in _______________ to, and was understood to the best of my knowledge by, the participant and that he/she has freely given their consent to participate* in the presence of the above named impartial witness (where applicable).

Printed name of person obtaining consent	Signature of person obtaining consent	Date

[*Only required if the participant is unable to read or write.]

A copy of this informed consent document has been provided to the participant.

Centre Number:
Study Number:
Participant Identification Number:

https://mc.manuscriptcentral.com/bmjpo

[Informed Consent for Participant with Impartial witness_26.11.19_v2]

Confidential: For Review Only

BMJ Paediatrics Open

Assessment of the role of gut health in childhood stunting in a multi-site, longitudinal study in India, Indonesia, and Senegal: a UKRI GCRF Action Against Stunting Hub protocol

Journal:	*BMJ Paediatrics Open*
Manuscript ID	bmjpo-2022-001637.R2
Article Type:	Protocol
Date Submitted by the Author:	29-Dec-2022
Complete List of Authors:	Momo Kadia, Benjamin; Liverpool School of Tropical Medicine Department of Clinical Sciences<br>Ramsteijn, Anouschka; University of Aberdeen<br>Dasi, Teena; National Institute of Nutrition<br>Fahmida, Umi; SEAMEO Regional Center for Food and Nutrition<br>Kulkarni, Bharati; National Institute of Nutrition<br>Faye, Babacar; Cheikh Anta Diop University of Dakar<br>Htet, Min Kyaw; SEAMEO Regional Center for Food and Nutrition<br>Sow, Doudou; Université Gaston Berger<br>Kalashikam, Rajender Rao; National Institute of Nutrition<br>Sharma, Ritu; National Institute of Nutrition<br>Sudibya, Arienta R.P.; SEAMEO Regional Center for Food and Nutrition<br>Kusuma, Sari; SEAMEO Regional Center for Food and Nutrition<br>Angelin, Tiffany C.; SEAMEO Regional Center for Food and Nutrition<br>Nurfadilah, Mifa; SEAMEO Regional Center for Food and Nutrition<br>Jobarteh, Modou; London School of Hygiene & Tropical Medicine<br>Diop, Ndeye Sokhna; Cheikh Anta Diop University of Dakar<br>Gabain, Isobel; University of London, Department of Pathobiology and Population Sciences<br>Calvo-Urbano, Beatriz; University of London, Department of Pathobiology and Population Sciences<br>Ferguson, Elaine; London School of Hygiene & Tropical Medicine<br>Haggarty, Paul; University of Aberdeen<br>Heffernan, Claire; London School of Hygiene & Tropical Medicine; London International Development Centre<br>Webster, Joanne P.; University of London, Department of Pathobiology and Population Sciences<br>Walker, Alan; University of Aberdeen<br>Allen, Stephen; Liverpool School of Tropical Medicine Department of Clinical Sciences
Keywords:	Gastroenterology, Growth, Microbiology, Molecular Biology, Parasitology

I, the Submitting Author has the right to grant and does grant on behalf of all authors of the Work (as defined in the below author licence), an exclusive licence and/or a non-exclusive licence for contributions from authors who are: i) UK Crown employees; ii) where BMJ has agreed a CC-BY licence shall apply, and/or iii) in accordance with the terms applicable for US Federal Government officers or employees acting as part of their official duties; on a worldwide, perpetual, irrevocable, royalty-free basis to BMJ Publishing Group Ltd ("BMJ") its licensees and where the relevant Journal is co-owned by BMJ to the co-owners of the Journal, to publish the Work in this journal and any other BMJ products and to exploit all rights, as set out in our licence.

The Submitting Author accepts and understands that any supply made under these terms is made by BMJ to the Submitting Author unless you are acting as an employee on behalf of your employer or a postgraduate student of an affiliated institution which is paying any applicable article publishing charge ("APC") for Open Access articles. Where the Submitting Author wishes to make the Work available on an Open Access basis (and intends to pay the relevant APC), the terms of reuse of such Open Access shall be governed by a Creative Commons licence – details of these licences and which Creative Commons licence will apply to this Work are set out in our licence referred to above.

Other than as permitted in any relevant BMJ Author's Self Archiving Policies, I confirm this Work has not been accepted for publication elsewhere, is not being considered for publication elsewhere and does not duplicate material already published. I confirm all authors consent to publication of this Work and authorise the granting of this licence.

Confidential: For Review Only

Assessment of the role of gut health in childhood stunting in a multi-site, longitudinal study in India, Indonesia, and Senegal: a UKRI GCRF Action Against Stunting Hub protocol

Benjamin Momo Kadia[1], Anouschka S. Ramsteijn[2], Teena Dasi[3], Umi Fahmida[4], Bharati Kulkarni[3], Babacar Faye[5], Min Kyaw Htet[4], Doudou Sow[6], Rajender Rao Kalashikam[3], Ritu Sharma[3], Arienta R.P. Sudibya[4], Sari Kusuma[4], Tiffany C. Angelin[4], Mifa Nurfadilah[4], Modou L. Jobarteh[7], Ndeye Sokhna Diop[5], Isobel Gabain[8], Beatriz Calvo-Urbano[8], Elaine Ferguson[7], Paul Haggarty[2], Claire Heffernan[7,8,9], Joanne P. Webster[8 α], Alan W. Walker[2α], Stephen J Allen[1α]

[α]Contributed equally

Authors' affiliations

1. Department of Clinical Sciences, Liverpool School of Tropical Medicine (LSTM), Liverpool, United Kingdom (UK)
2. Rowett Institute, University of Aberdeen, Aberdeen, UK
3. ICMR-National Institute of Nutrition, Hyderabad, India
4. Southeast Asian Ministry of Education Organisation Regional Centre for Food and Nutrition (SEAMEO RECFON), East Jakarta, Indonesia
5. Service de Parasitologie-Mycologie, Faculté de Médecine, Université Cheikh Anta Diop (UCAD), Dakar, Sénégal
6. Service de Parasitologie-Mycologie, UFR Sciences de la Santé, Université Gaston Berger, Saint Louis, Sénégal
7. London School of Hygiene and Tropical Medicine (LSHTM), London, UK
8. Department of Pathobiology and Population Sciences, Royal Veterinary College, University of London, London, UK
9. London International Development Centre (LIDC), London, UK

Authors' emails

BMK: benjamin.kadia@lstmed.ac.uk; ASR: anouschka.ramsteijn@abdn.ac.uk; TD: teenarajeev@rediffmail.com; UF: umifahmida@gmail.com; BK: dr.bharatikulkarni@gmail.com; BF: babacar2.faye@ucad.edu.sn; MKH: kyawhtet@gmail.com; DS: doudou.sow@ugb.edu.sn; RRK: rkrajender@yahoo.com, RS: jnb.ritusharma@gmail.com, ARPS: arientasudibya@gmail.com; SK: skusuma@seameo-recfon.org; TCA: tiffany.angelin@gmail.com; MN: mifanurfadilah@gmail.com; MLJ: modou.jobarteh@lshtm.ac.uk; NSD: yayoumbayediop@gmail.com; IG: igabain19@rvc.ac.uk; BC: bcalvo@rvc.ac.uk; EF: elaine.ferguson@lshtm.ac.uk; PH:paul.haggarty@abdn.ac.uk; CH: Claire.Heffernan@lidc.ac.uk; JPW: jowebster@rvc.ac.uk; AWW: alan.walker@abdn.ac.uk; SJA: stephen.allen@lstmed.ac.uk;

Corresponding author: Benjamin Momo Kadia: Department of Clinical Sciences, LSTM; email: benjamin.kadia@lstmed.ac.uk

ABSTRACT

Introduction: Childhood stunting has a complex aetiology, with poor gut health being an important contributor. This study will assess inter-relationships between maternal and infant gut health indices and infant linear growth. Inter-relationships between gut health indices, systemic inflammation, and growth hormones in early childhood will also be assessed.

Methods and analysis: A longitudinal observational study of cohorts of 600 newborns and their mothers in India, Indonesia, and Senegal will be conducted. Women will be recruited during pregnancy and their children followed up to age 24 months. Stool, urine, and blood samples will be collected from the women and children for assessments of helminthic and protozoal parasites, bacterial pathogens, faecal microbiota taxa, biomarkers of environmental enteric dysfunction, systemic inflammation, and growth hormones. Child anthropometric measurements will be collected at birth and at ages 3, 6, 9, 12, 18 and 24 months. The gut health indices will be integrated with cohort data from other Action Against Stunting Hub (AASH) workstreams for interdisciplinary analyses of childhood stunting and the development of a new typology of stunting.

Discussion: This study will advance scientific understanding of the role of gut health in childhood stunting and will contribute to a broader knowledge of the complex aetiology of this condition as part of the interdisciplinary AASH research to reduce the global burden of childhood stunting.

Ethics and dissemination: This study has been approved by the relevant Ethics Committees in Senegal, India, and Indonesia and LSHTM. The results will be submitted for publication in peer-reviewed journals.

Confidential: For Review Only

SUMMARY BOX

What is known about the subject?

- In areas with poor hygiene and sanitation, recurrent enteric infections and alterations in the gut microbiota are associated with environmental enteric dysfunction (EED).
- EED is an important precursor of childhood stunting.

What this study hopes to add

- Compare characteristics of gut health in mothers and their infants and elucidate their association with childhood stunting across three distinct geographical settings, with the inclusion of mothers being a novel approach providing more robust evidence on early life determinants of childhood stunting.
- Contribute to the development of a new typology of childhood stunting based on knowledge of the relationship between the gut and other host- and environmental factors, and to support development of interdisciplinary approaches to prevent and reduce childhood stunting.

How this study might affect research, practice, or policy

- Identify biomarkers of gut health that may serve as outcome measures in future interventional studies and inform the development of diagnostic and management options for EED in resource-constrained settings.

Confidential: For Review Only

ABBREVIATIONS

AASH	Action Against Stunting Hub
AGP	α_1-acid glycoprotein
CRP	C-reactive protein
EED	Environmental enteric dysfunction
IFABP	Intestinal fatty acid binding protein
IGF-1	Insulin-like growth factor-1
IGFBP3	Insulin-like growth factor binding protein 3
LAZ	Length-for-age z-score

Confidential: For Review Only

INTRODUCTION

Worldwide, more than 1 in 5 children under five years of age are currently stunted, with more than half residing in Asia and two out of five in Africa [1]. Childhood stunting is defined as having a height-for-age z-score more than two standard deviations below the median of the WHO child growth standards [2]. Whilst the aetiology of stunting is complex, several studies suggest that it is associated with environmental enteric dysfunction (EED) resulting from asymptomatic gut infection with pathogenic microbes [3,4].

EED refers to the abnormal gut structure and function commonly found in people living in environments with poor sanitation and hygiene, with onset detected as early as 12 weeks after birth despite exclusive breast feeding [5,6]. EED is characterised by villous atrophy, crypt hyperplasia, chronic enteric inflammation, and increased gut mucosal permeability. It leads to reduced nutrient absorption, increased risk of systemic infection due to reduced integrity of the gut barrier, particularly due to gut-derived Gram-negative bacteria, and impaired immune responses to oral vaccines [5,6]. EED contributes to stunting through nutrient malabsorption and systemic inflammation, with reduced production of growth hormones [5–7]. Furthermore, EED has been associated with disruption in the ecological niches that support the gut microbiota [5]. It is known that malnutrition and growth faltering in early life are associated with microbiota immaturity [8], and experimental evidence suggests that the gut microbiota may be causally involved in the manifestation of stunting [9]. The gut microbiota also plays an important role in protecting against colonisation and invasion of the gut by pathogens [6].

Previous studies of stunting have explored various biomarkers of EED, including plasma α_1-acid glycoprotein (AGP, a longer-term marker of systemic inflammation) [7,10–15], faecal myeloperoxidase (MPO, a marker of intestinal inflammation) [11–13,16], faecal α_1-antitrypsin (AAT, a marker of protein loss and intestinal permeability) [11,13,16] and plasma intestinal fatty acid binding protein (IFABP, a marker of mucosal permeability and gut integrity) [7,10,16,17], and also measured plasma growth hormones (insulin-like growth factor (IGF)-1 and its binding protein IGFBP3) [7,10,12]. However, a consensus has not been reached on the biomarkers to use as standard for diagnosis and monitoring of EED. Thus, in low-income settings where EED and stunting are most prevalent, the association of biomarkers of EED with linear growth requires further investigation.

In this study, we will assess aspects of gut health, including gut microbiota profiles, colonisation of the gut by pathogenic microbes (bacterial and parasitic pathogens), and biomarkers of EED and associated pathology (systemic inflammation and reduced growth hormones) during pregnancy and early childhood, and their role in childhood stunting.

Confidential: For Review Only

METHODS AND ANALYSIS

Study design and population

This is a longitudinal, observational study which is part of the UKRI GCRF Action Against Stunting Hub (AASH). Full details of the overall AASH design are available elsewhere (see the overview of study design, data collection and management procedures of the AASH in the supplement), but details relating to the Gut Health component workstream are summarised below.

Women in the second and third trimesters of pregnancy will be recruited in Hyderabad in India (urban), East Lombok in Indonesia (rural), and Kaffrine District in Senegal (rural), and their newborns will be followed up to age 24 months. These three areas were chosen because they have high proportions of stunted growth. In India, 30.9% of children under five are stunted [1] and Hyderabad is among the highest burden districts, bearing up to 11.6% of all stunted children under five in the country [18]. In Indonesia, 31.8% of children under five are stunted [1], with East Lombok having one of the highest subnational rates of about 44% [19]. Despite having a lower prevalence (17.2%), Senegal is still off-track to achieve a 50% reduced prevalence of stunted growth by 2030 [1]. In Kaffrine district, up to 26% of children under five are stunted, which exceeds the national prevalence [20].

For recruitment purposes, the sampling frame (lists of pregnant women) will be obtained from health services in each country. From the lists, 50 women will be randomly selected each month and those who meet the inclusion criteria will be recruited. If women from this random selection do not fit the inclusion criteria, women will be selected from the rest of the initial lists for that month.

Sample size determination

Sample size calculations were performed in R with the package “pwr” and were based on a multiple linear regression analysis with 10 predictors, an effect size of 0.035, power of 80%, significance level of 0.05 and drop-out rate of 20%. These calculations resulted in a total of 592 participants per site.

Study procedures

Sample collection

Stool samples will be collected from pregnant women in the third trimester. In infants/children, stool samples will be collected either at a health facility or in the participants’ homes at ages 1 month, 6 months and 24 months. For stool samples collected at home, study staff will visit either on the day before or on the morning of sample collection to provide the mother/carer with a disposable nappy or plastic sheet, sample collection containers with spatula, plastic

Confidential: For Review Only

specimen bag, small cold box with ice pack, and a bar of soap. The staff will explain the sample collection procedure to the mother/carer through verbal and pictorial information, and the staff will check the procedure is understood. Mothers/carers will be asked to snugly fit a nappy or plastic sheet to the infant last thing at night and collect the next stool produced by the infant, either by pouring it from the nappy/sheet if the sample is loose, or using the spatula if it is formed, and fill the sample containers. They will place the filled container in the specimen bag and then in the cold box and wash their hands with soap. Field staff will return later the same day or the following day to label the sample and transfer it to the laboratory. Subject to agreement with the mother/carer, this process will be repeated over the following fourteen days until an adequate stool sample is obtained (at least 5 ml of liquid stool or 5 grams of formed stool). To avoid dilution of biomarkers in children suffering from acute diarrhoea, stool collection will be delayed until the stools have returned to their usual consistency. Based on the WHO definition [21], acute diarrhoea will be considered as significantly more frequent or loose/watery stools in the mother's/carer's opinion in exclusively breastfed babies and three or more liquid stools in the last 24 hours in children receiving other feeds. Mothers/carers will be fully briefed on recognising acute diarrhoea as distinct from loose stools within exclusively breast-fed babies. During the visits to collect samples at age 6 and 24 months, trained research staff will collect 2.5 ml venous blood. Subject to the agreement of the mother/carer, efforts will be made to limit attempts to collect the required blood volume to three. Finally, early morning urine samples will be collected from pregnant mothers and infants at 6 and 24 months in Senegal to identify the presence and intensity of *Schistosoma haematobium* and hybrids therein. All samples will be transported to laboratories in a cold box. The time between sample collection and receipt in the laboratory will be recorded. Pilot testing of sample collection processes was conducted in each country.

Anthropometric measurements (including length and weight) will be taken in duplicate from infants at birth, 3, 6, 9, 12 18 and 24 months of age as described in detail elsewhere in the supplement (Anthropometric, biochemical, dietary, morbidity and wellbeing assessment in women and children in Indonesia, India, and Senegal: a UKRI GCRF Action Against Stunting Hub protocol). Z-scores for length-for-age (LAZ) will be calculated using the 2006 WHO Growth Standards and stunted growth will be defined as a LAZ <-2 standard deviations [2].

Laboratory analysis

Approximately 1 gram of faecal material will be aliquoted into a DNA/RNA Shield Faecal Collection Tube containing a preservative buffer solution (Zymo Research, California, USA) for DNA extraction using the ZymoBIOMICS DNA Miniprep kit (Zymo Research, California, USA) for subsequent microbiome and qPCR-based analyses. Stool samples and aliquots in DNA/RNA Shield will be stored at -20°C until further processing.

Confidential: For Review Only

Kato-Katz thick smear microscopy (duplicate slides per single stool) will be performed on fresh (untreated) stool to obtain individual egg counts of key helminth parasites reported and/or predicted to be associated with physical and/or cognitive childhood stunting (e.g. *Ascaris* spp., hookworm, *Trichuris trichiura, Hymenolepis nana*) [22]. Slides will be read within 60 minutes post Kato-Katz preparation to obtain hookworm data. The slides will then be maintained at room temperature prior to microscopic examination of the remaining parasites present. The amount of stool placed on the Kato Katz slide is 41.7 mg, so the numerical values obtained will be multiplied by 24 to give the number of eggs per gram (epg)/Faecal Egg Count (FEC) – the standard measurement to assess the intensity of infection.

Multi-parallel qPCR on DNA extracted from faeces will be performed for high sensitivity and specificity identification of key gastrointestinal parasites [23], based on a modified protocol with updated primers and quality control [24,25]. This is a high throughput, species-specific molecular diagnostic tool for routine surveillance, monitoring and research, which allows more accurate identification and quantification of key pathogens associated with physical and cognitive stunting in children relative to slide-based microscopy alone [24,25]. Infection levels can be more precisely measured in low prevalence settings where intensities are low (as predicted in infants). This will target *Ascaris lumbricoides*, *Ancylostoma duodenale*, *Necator americanus*, *Trichuris trichiura*, *Strongyloides stercoralis*, and protozoans such as *Giardia lamblia*, *Cryptosporidium parvum/hominis* and *Entamoeba histolytica/dispar*.

Multiplex PCR will also be used to identify pathogenic strains of *Escherichia coli* (*E. coli*): enteropathogenic *E. coli*, enteroaggregative *E. coli*, enterotoxigenic *E. coli*, and enteroinvasive *E. coli* using methods and primers developed for the MAL-ED study [26]. In addition, analysis of the faecal microbiota (see below) may also putatively identify potential enteropathogens at the genus level, such as *Helicobacter, Campylobacter*, *Plesiomonas*, *Yersinia*, *Aeromonas, Shigella/Escherichia,* and *Salmonella* spp..

For microbial culture to detect *Salmonella* spp. and *Shigella* spp., swabbing onto agar plates or inoculating approximately 1 g of fresh stool in Selenite broth for enrichment followed by culture in selective media MacConkey Agar and Xylose Lysine Deoxycholate Agar (MCA and XLD) or by automated culture in Senegal will be done immediately after samples are brought to the laboratory. Gram staining and biochemical tests such as Kligler Iron Agar (KIA), Motility Indole Ornithine (MIO), Lysine decarboxylase (LDC), Urea and Oxidase will be used for identifying the cultured isolates at the genus level, complemented by serological tests to identify the bacterial species using BD Difco Salmonella O Antiserum Poly A-I and BD Difco Shigella Antiserum Poly Group A, A1, B, C, C1, C2 and D.

Myeloperoxidase and alpha 1-antitrypsin will be measured in stool samples using the Immunodiagnostik AG (Bensheim, Germany) ELISA Kit. Sample preparation and analysis will be as per manufacturer's instructions.

Microbiota profiling will be done by 16S rRNA gene amplicon sequencing. We will use barcoded primers from the Earth Microbiome Project protocol [27], with slight modifications to increase detected diversity [28], to amplify the V4 region of the 16S rRNA gene using PCR on DNA extracted from stool samples. 16S rRNA gene amplicon sequencing will be carried out using the Illumina MiSeq platform. For amplicons from Indonesia and Senegal this will be carried out by sequencing providers in the UK, while sequencing of Indian sample amplicons will take place at a sequencing provider in India. Negative (no DNA template "blanks") and positive (extracted DNA and 16S rRNA gene amplicons from one benchmark stool sample) control samples will be included in each sequencing run for quality control purposes, and to ensure consistency between runs, both longitudinally, and between participant countries.

For the urine samples in Senegal, assessment for blood in urine will be performed immediately upon receipt in the laboratory (Hemastix™ Reagent Strip; Bayer Corp, Diagnostics Div., New York, USA). Standard urine filtration and microscopy (duplicate 10 ml per urine sample) will be performed to identify and quantify *Schistosoma haematobium* (and hybrids therein) infection status and recorded as eggs per 10 ml/urine. Positive filters will be placed in fresh water and exposed to light to facilitate egg-hatching into miracidia. Free-swimming miracidia will be individually pipetted onto Whatman Indicating FTA Classic cards (GE Healthcare Life Sciences, UK) for DNA storage and subsequent molecular analysis. Individual *Schistosoma* DNA extracts will be characterised by amplification of a partial fragment of the mitochondrial cytochrome c oxidase subunit 1 (*cox1*) and the complete nuclear ribosomal DNA internal transcribed spacer (ITS) to identify species/species-combination, following protocols described elsewhere [29].

Blood samples will be centrifuged and separated on receipt at the laboratory and plasma stored at -80°C until analyses. Samples in Senegal will be stored at -20°C and transported to Dakar within days of collection to store at -80°C. Blood samples will be analysed for biomarkers of inflammation (AGP; Human AGP Quantikine ELISA Kit) and C Reactive Protein (CRP; Human CRP/CRP Quantikine ELISA kit in India and Senegal; Quansys multiplex in Indonesia); gut mucosal integrity (IFABP; Human FABP2/I-FABP Quantikine ELISA Kit in India and Senegal; Quansys multiplex in Indonesia) and plasma growth hormones (IGF-1 and IGF BP3; Human IGF-1 and IGFBP-3 Quantikine ELISA Kits respectively, R&D Systems, Inc. Minneapolis, USA in India and Senegal and Quansys multiplex in Indonesia). Assays will be carried out according to the manufacturers' instructions at the National Institute of Nutrition, Hyderabad in India and the Université Cheikh Anta DIOP, Dakar in Senegal. In Indonesia, sample preparation will be done in Lombok and analyses in SEAMEO RECFON's laboratory in Jakarta.

All participants with clinically relevant bacterial or parasitic infections will be offered treatment. After these analyses, sample aliquots will be stored at -80°C.

Confidential: For Review Only

Data management and analysis

Data management will follow procedures established by the AASH (see the overview of study design, data collection and management procedures of the AASH). This includes a data sharing strategy that will build upon the guiding principles for scientific management and stewardship and Concordat on Open Research Data to ensure equitable access to data.

For microbiota analysis, FASTQ files containing 16S rRNA gene amplicon sequences will be processed using well validated software pipelines such as mothur [30] or DADA2 [31]. These pipelines include various quality control steps and will produce final output tables containing the proportional abundances of various microbiota taxa in each sample. They can also generate various metrics of bacterial diversity within and between samples. These data will be visualised in the form of dendrograms, ordination plots, heatmaps and box plots. Microbiota 16S rRNA gene sequence data will be released publicly via the European Nucleotide Archive at the end of the study.

Multi-level models taking into consideration aggregate units such as geographical setting will be used to investigate associations between enteropathogens/gut parasites, biomarker concentrations and LAZ, to assess longitudinal associations between biomarker concentrations and growth rates, and to determine which biomarkers, enteropathogens and/or characteristics of the gut microbiota are associated with stunted growth at specific timepoints. In addition, Bayesian methods, using Directed Acyclic Graphs, will be used to investigate a variety of possible interactions between gut health indices and linear growth (stunting) and to assess combinatorial effects of the diverse elements of gut health (expressed as categorical or continuous variables, as appropriate) on stunting. When the data being analysed include repeated measurements from the same individual, random intercept models will be employed. The number of missing data for each variable of interest will be reported and the distributions of key variables will be tabulated to compare individuals with and without complete data. Analyses will be carried out both on datasets where multiple imputation will be implemented, and on datasets that will be restricted to complete cases.

To allow for interdisciplinary analyses and the development of the typology of stunting, multi-level modelling and Bayesian methods will be extended to integrate cohort data collected in other workstreams. Stata version 17 and other appropriate software will be used for the analysis.

Patient and public involvement

There was no involvement of patients or the public in the design of this study.

Confidential: For Review Only

DISCUSSION

The limited impact of interventions to prevent or ameliorate stunting in young children in low-resource settings has resulted in a renewed interest in the role of gut health. The AASH cohorts will be amongst the largest investigating the inter-relationships between gut colonisation with bacterial, protozoal and helminth pathogens, the gut microbiota, and biomarkers of EED, and their impact on linear growth in populations with different prevalence rates of stunting (moderate to high) and environmental conditions.

The AASH offers the opportunity to compare gut health characteristics and their association with stunting across three distinct geographical settings. This is particularly important as there is limited evidence to date regarding the role of gut health in children in West Africa and Indonesia. In addition, our findings will be integrated with data collected in other AASH workstreams to advance scientific understanding of how gut health interacts with other host- and environmental factors that underlie common typologies of stunting and increase the evidence base for future intervention studies. This evidence base will be further strengthened by the investigation of elements of maternal gut health as novel drivers of poor gut health and stunted growth in infants. Harmonised procedures to collect, manage and analyse data and samples across the countries will greatly enhance the validity of our findings.

In low-income settings, there is a need to further investigate biomarkers of intestinal structure and function to identify acceptable and affordable tools that can be scaled-up at the population level to diagnose and monitor EED. Further, it is of interest to identify possible geographic-specific and seasonal variations in the association between these biomarkers and stunting. The selection of biomarkers for this study was informed by previous reports of their utility in reflecting the aetiopathogenesis of EED and association with linear growth faltering [10, 12, 13, 15, 16].

This study will have some limitations. Although our study design allows us to explore similarities and differences across cohorts from three countries, our findings may not be generalisable to gut health and growth in other low-resource settings where the intensity of infection, characteristics of the gut microbiota and pathology of EED may differ. In general, it is a challenge to move from associations to an understanding of causality in an observational study. However, the SENeGal SYNbiotic (SENGSYN) study, a clinical trial nested within the AASH investigating the effect of supplementation with synbiotics on growth and biomarkers of gut health (see supplement protocol entitled ‘Improving gut health and growth in early life: a protocol for an individually randomised, two-arm, 2 open-label, controlled trial of a synbiotic in infants in Kaffrine District, Senegal’), may build on associations between gut health and growth determined in the observation cohorts to provide evidence of causality. From a technical perspective, our method of stool collection using cold boxes and minimising time between sample provision and processing in the laboratories is pragmatic. However, we cannot exclude

Confidential: For Review Only

the possibility that changes in stool biomarkers, pathogens and the microbiota may occur during transfer. We will, however, assess whether time between collection and receipt in the laboratory is associated with the results of the various analyses. We also acknowledge that providing a picture-based reference chart for mothers/carers to assess stool consistency would be an even more robust approach to assess stool consistency for the exclusion of diarrhoeal samples. Nonetheless, as part of quality control, trained laboratory technicians will cross-reference all collected samples to ensure they are of appropriate consistency before being further processed. Furthermore, we acknowledge that the WHO Child Growth Standards do not necessarily provide a good statistical fit to each country, but these internationally approved standards are widely accepted and facilitate cross-country comparisons, which is considered particularly important when including the three countries in this study. Previous multi-country studies notably, the MAL-ED study[2], have used the same growth standards.

CONCLUSIONS

The findings of this study will improve scientific understanding of how gut health contributes to stunting, either directly, or through inter-links with the wide range of host- and environmental factors (including maternal gut health characteristics) monitored in the AASH project, and how these may vary between different geographic locations. Our findings will inform the development of diagnostic and management options for EED and identify biomarkers of gut health that may serve as outcome measures in future intervention trials in resource-constrained settings.

https://mc.manuscriptcentral.com/bmjpo

Confidential: For Review Only

REFERENCES

1. World Health Organisation (WHO), World Bank, United Nations International Children's Emergency Fund. Levels and trends in child malnutrition: Key findings of the 2021 Edition of the Joint Child Malnutrition Estimates. Available at https://www.who.int/publications-detail-redirect/9789240025257

2. WHO Multicentre Growth Reference Study Group. WHO Child Growth Standards based on length/height, weight and age. Acta Paediatr Suppl. Norway; 2006;450:76–85. doi.10.1111/j.1651-2227.2006.tb02378.x

3. Owino V, Ahmed T, Freemark M, et al. Environmental enteric dysfunction and growth failure/stunting in global child health. Pediatrics. 2016; 38(6): e20160641. doi: 10.1542/peds.2016-0641

4. Nataro JP, Guerrant RL. Chronic consequences on human health induced by microbial pathogens: Growth faltering among children in developing countries. Vaccine. 2017;35:6807–6812. doi.10.1016/j.vaccine.2017.05.035

5. Tickell KD, Atlas HE, Walson JL. Environmental enteric dysfunction: A review of potential mechanisms, consequences and management strategies. BMC Med. 2019;17:1–9. doi.10.1186/s12916-019-1417-3

6. Naylor C, Lu M, Haque R, et al. Environmental Enteropathy, Oral Vaccine Failure and Growth Faltering in Infants in Bangladesh. EBioMedicine. 2015;2:1759–1766. doi.10.1016/j.ebiom.2015.09.036

7. Prendergast AJ, Rukobo S, Chasekwa B, et al. Stunting is characterized by chronic inflammation in zimbabwean infants. PLoS One. 2014;9:e86928. doi.10.1371/journal.pone.0086928

8. Subramanian S, Huq S, Yatsunenko T, et al. Persistent gut microbiota immaturity in malnourished Bangladeshi children. Nature. 2014;510:417–21. doi.10.1038/nature13421

9. Blanton L V, Charbonneau MR, Salih T, et al. Gut bacteria that prevent growth impairments transmitted by microbiota from malnourished children. Science. 2016;351:854. doi. 10.1126/science.aad3311

10. Arndt MB, Cantera JL, Mercer LD, et al. Validation of the Micronutrient and Environmental Enteric Dysfunction Assessment Tool and evaluation of biomarker risk factors for growth faltering and vaccine failure in young Malian children. PLoS Negl Trop Dis. 2020;14:e0008711. doi.10.1371/journal.pntd.0008711

11. Campbell RK, Schulze KJ, Shaikh S, et al. Environmental enteric dysfunction and systemic inflammation predict reduced weight but not length gain in rural Bangladeshi children. Br J Nutr. 2018; ;119(4):407-414. doi: 10.1017/S0007114517003683

12. Iqbal NT, Sadiq K, Syed S, et al. Promising Biomarkers of Environmental Enteric Dysfunction: A Prospective Cohort study in Pakistani Children. Sci Rep. 2018;8:2966. doi.10.1038/s41598-018-21319-8

Confidential: For Review Only

13. Kosek MN, Ahmed T, Bhutta ZA, et al. Causal Pathways from Enteropathogens to Environmental Enteropathy: Findings from the MAL-ED Birth Cohort Study. EBioMedicine. 2017;18:109–117. doi.10.1016/j.ebiom.2017.02.024

14. Merrill RD, Burke RM, Northrop-Clewes CA, et al. Factors associated with inflammation in preschool children and women of reproductive age: Biomarkers Reflecting Inflammation and Nutritional Determinants of Anemia (BRINDA) project. Am. J. Clin. Nutr. 2017. doi.10.3945/ajcn.116.142315

15. Lauer JM, Ghosh S, Ausman LM, et al. Markers of Environmental Enteric Dysfunction Are Associated with Poor Growth and Iron Status in Rural Ugandan Infants. J Nutr. 2020;150:2175–82. doi: 10.1093/jn/nxaa141

16. Guerrant RL, Leite AM, Pinkerton R, Medeiros PHQS, Cavalcante PA, DeBoer M, et al. Biomarkers of environmental enteropathy, inflammation, stunting, and impaired growth in children in northeast Brazil. PLoS One. 2016;11:e0158772. doi.10.1371/journal.pone.0158772

17. Zambruni M, Ochoa TJ, Somasunderam A, et al. Stunting Is Preceded by Intestinal Mucosal Damage and Microbiome Changes and Is Associated with Systemic Inflammation in a Cohort of Peruvian Infants. Am J Trop Med Hyg. 2019;101:1009–17. doi.10.4269/ajtmh.18-0975

18. Ministry of Health and Family Welfare, India. National Family Health Survey (NFHS-5) State Factsheets (2019-2020). Available at http://rchiips.org/nfhs/factsheet NFHS-5.shtml

19. Indonesia Ministry of Health. National Institute of Health Research and Development. Indonesia Basic Health Research (RISKESDAS) 2018. Available at http://labmandat.litbang.depkes.go.id/images/download/laporan/RKD/2018/Laporan%7B%5C_%7DNasional%7B%5C_%7DRKD2018%7B%5C_%7DFINAL.pd

20. National Agency for Statistics and Demography - ANSD / Senegal and ICF. Demographic and Continuous Health Survey (EDS-Continue) 2017. Dakar, Senegal: ANSD and ICF; 2018. Available from: http://dhsprogram.com/pubs/pdf/FR345/FR345.pdf.

21. World Health Organisation (WHO). WHO Fact sheets: Diarrhoeal disease (updated 2 May 2017). Available at https://www.who.int/news-room/fact-sheets/detail/diarrhoeal-disease

22. Raj, E., Calvo-Urbano, B., Heffernan, C. *et al.* Systematic review to evaluate a potential association between helminth infection and physical stunting in children. *Parasites Vectors*. 2022; 15, 135. doi.org/10.1186/s13071-022-05235-5

23. Easton A V, Oliveira RG, O'Connell EM, et al. Multi-parallel qPCR provides increased sensitivity and diagnostic breadth for gastrointestinal parasites of humans: field-based inferences on the impact of mass deworming. Parasit Vectors. 2016;9:38. doi.10.1186/s13071-016-1314-y

24. Mejia R, Vicuña Y, Broncano N, et al. A novel, multi-parallel, real-time polymerase chain reaction approach for eight gastrointestinal parasites provides improved diagnostic capabilities

Confidential: For Review Only

to resource-limited at-risk populations. Am J Trop Med Hyg. 2013/03/18. The American Society of Tropical Medicine and Hygiene; 2013;88:1041–7. doi.10.4269/ajtmh.12-0726

25. Papaiakovou M, Gasser RB, Littlewood DTJ. Quantitative PCR-Based Diagnosis of Soil-Transmitted Helminth Infections: Faecal or Fickle? Trends Parasitol. 2019;35:491–500. doi.10.1016/j.pt.2019.04.006

26. Houpt E, Gratz J, Kosek M, et al. Microbiologic methods utilized in the MAL-ED cohort study. *Clin Infect Dis*. 2014;59 Suppl 4(Suppl 4): S225-S232. doi:10.1093/cid/ciu413

27. Caporaso JG, Lauber CL, Walters WA, et al. Global patterns of 16S rRNA diversity at a depth of millions of sequences per sample. Proc Natl Acad Sci U S A. 2011;108 Suppl:4516–22. doi.10.1073/pnas.1000080107

28. Walters W, Hyde ER, Berg-Lyons D, et al. Improved Bacterial 16S rRNA Gene (V4 and V4-5) and Fungal Internal Transcribed Spacer Marker Gene Primers for Microbial Community Surveys. mSystems. 2016;1:e00009-15. doi.10.1128/mSystems.00009-15

29. Léger E, Borlase A, Fall CB, et al. Prevalence and distribution of schistosomiasis in human, livestock, and snail populations in northern Senegal: a One Health epidemiological study of a multi-host system. Lancet Planet Heal. 2020;4:e330–42. doi.10.1016/S2542-5196(20)30129-7

30. Schloss PD, Westcott SL, Ryabin T, et al. Introducing mothur: open-source, platform-independent, community-supported software for describing and comparing microbial communities. Appl Environ Microbiol. 2009;75:7537–41. doi.10.1128/AEM.01541-09

31. Callahan BJ, McMurdie PJ, Rosen MJ, et al. DADA2: High-resolution sample inference from Illumina amplicon data. Nat Methods. 2016;13:581–3. doi.10.1038/nmeth.3869

FOOTNOTES

Contributors

BMK: Clinical Research Associate dedicated to the overview and delivery of the Gut Health workstream of the AASH; drafted the initial manuscript.

ASR: Provides research and organisational support in the microbiome component of the study; critically revised the manuscript.

TD: Theme lead for gut enteropathogens and parasitology, India; lead for the development of standard operating procedures.

UF: Country lead, AASH, Indonesia; contributed to study design and coordination of the study and development of standard operating procedures.

BK: Country lead, AASH, India; contributed to the design and coordination of the study in India.

BF: Country lead, AASH, Indonesia, and contributed to the design of the study and coordinates the study in Senegal.

Confidential: For Review Only

MKH: Gut Health theme lead, Indonesia; contributes to the implementation of the study in Indonesia.

DS: Gut Health theme lead, Senegal; oversees the implementation of the study in Senegal.

RRK: supports coordination of laboratory analysis in India; reviewed the manuscript

RS: field work support and data collection in India; reviewed the manuscript

ARPS, SK, TCA and MN: Actively contribute to the set-up and implementation of the AASH in Indonesia.

MLJ: Edited the manuscript; reviewed the manuscript for technical and intellectual consistency

NSD: Participates in the set-up and implementation of the study in Senegal.

IG: Provided research and organisational support in the parasitology aspect of the study

BC: Provided guidance on preparationof data management and statistical analysis plan for the Gut Health workstream

EF: Oversees the Nutrition/Anthropometry workstream of the Stunting Hub; contributed to the design of the study, and critically revised the protocol.

PH and CH: Contributed to the conception of the study and critically revised the protocol

JPW: Conception of the study; oversees the parasitology theme of the study; critically revised the protocol and manuscript.

AWW: Conception of the study, oversees the microbiome component of the study; critically revised the protocol and supervised drafting of the manuscript.

SJA: Conception of the study; oversees the delivery of the Gut Health workstream; critically revised the protocol and supervised drafting of the manuscript.

Funding: This work is supported by the London School of Hygiene and Tropical Medicine through funding from the UKRI GCRF Action Against Stunting Hub (grant number: MR/S013/13X/1). AWW and PH are supported by core funding from the Scottish Government's Rural and Environment Science and Analytical Services (RESAS) division (no grant number).

Competing interests: The authors declare that they have no competing interests.

Consent for publication: Not required.

Ethical considerations: This study has been approved by the ethics committees of the National Institute for Nutrition, India and the University of Indonesia, the National Ethics Committee for Scientific Research in Senegal, and the Research Ethics Committee of the London School of Hygiene and Tropical Medicine in the UK.

Data availability statement: No data are available. Data sharing is not applicable to this manuscript as it describes a protocol.

Confidential: For Review Only

LONDON SCHOOL of HYGIENE &TROPICAL MEDICINE

PARTICIPANT CONSENT FORM

Title of Project: UKRI GCRF Action against Stunting Hub – Observational Cohort Study - Mothers
Name of PI/Researcher responsible for project: Professor Claire Heffernan

Statement	Please initial or thumbprint* each box
I confirm that I have read the information sheet dated 26/11/2019 (version 2) for the above named study. I have had the opportunity to consider the information, ask questions and have these answered satisfactorily.<br>**OR**<br>I have had the information explained to me by study personnel in a language that I understand. I have had the opportunity to consider the information, ask questions and have these answered satisfactorily.	
I understand that my participation is voluntary and that I am free to withdraw at any time without giving any reason, without my/my baby's medical care or legal rights being affected.	
I understand that relevant sections of my/my baby's medical notes and data collected during the study may be looked at by authorised individuals from the study team, where it is relevant to my/my baby taking part in this research. I give permission for these individuals to have access to my records. I understand that they will only access information relevant to the birth of my baby.	
I understand that data about me/my baby may be shared via a public data store or by sharing directly with other researchers, and that I/my baby will not be identifiable from this information.	
I understand that the samples of blood, saliva, urine, hair, poo and breastmilk collected from me/my baby may be used to support other research in the future, and may be shared anonymously with other researchers, for their ethically-approved projects.	
I understand and agree that my words may be quoted in publications, reports, web pages, and other research outputs. I understand that I will not be named or in any way personally identifiable in these outputs.	
I understand that I may be contacted at a later date to consider participating in a separate study about my baby's health.	
I agree to take part in the above named study.	

Printed name of participant	Signature of participant	Date

Printed name of impartial witness*	Signature of impartial witness*	Date

I attest that I have explained the study information accurately in _______________ to, and was understood to the best of my knowledge by, the participant and that he/she has freely given their consent to participate* in the presence of the above named impartial witness (where applicable).

Printed name of person obtaining consent	Signature of person obtaining consent	Date

[*Only required if the participant is unable to read or write.]

A copy of this informed consent document has been provided to the participant.

Centre Number:
Study Number:
Participant Identification Number:

[Informed Consent for Participant with Impartial witness_26.11.19_v2]

Confidential: For Review Only
